# Respiration Gates Sensory Input Responses in the Mitral Cell Layer of the Olfactory Bulb

**DOI:** 10.1371/journal.pone.0168356

**Published:** 2016-12-22

**Authors:** Shaina M. Short, Thomas M. Morse, Thomas S. McTavish, Gordon M. Shepherd, Justus V. Verhagen

**Affiliations:** 1 Yale School of Medicine, Dept. Neuroscience, New Haven, CT, United States of America; 2 The John B. Pierce Laboratory, New Haven, CT, United States of America; Monell Chemical Senses Center, UNITED STATES

## Abstract

Respiration plays an essential role in odor processing. Even in the absence of odors, oscillating excitatory and inhibitory activity in the olfactory bulb synchronizes with respiration, commonly resulting in a burst of action potentials in mammalian mitral/tufted cells (MTCs) during the transition from inhalation to exhalation. This excitation is followed by inhibition that quiets MTC activity in both the glomerular and granule cell layers. Odor processing is hypothesized to be modulated by and may even rely on respiration-mediated activity, yet exactly how respiration influences sensory processing by MTCs is still not well understood. By using optogenetics to stimulate discrete sensory inputs *in vivo*, it was possible to temporally vary the stimulus to occur at unique phases of each respiration. Single unit recordings obtained from the mitral cell layer were used to map spatiotemporal patterns of glomerular evoked responses that were unique to stimulations occurring during periods of inhalation or exhalation. Sensory evoked activity in MTCs was gated to periods outside phasic respiratory mediated firing, causing net shifts in MTC activity across the cycle. In contrast, odor evoked inhibitory responses appear to be permitted throughout the respiratory cycle. Computational models were used to further explore mechanisms of inhibition that can be activated by respiratory activity and influence MTC responses. *In silico* results indicate that both periglomerular and granule cell inhibition can be activated by respiration to internally gate sensory responses in the olfactory bulb. Both the respiration rate and strength of lateral connectivity influenced inhibitory mechanisms that gate sensory evoked responses.

## Introduction

Olfaction relies on respiration to bring odor molecules to the nasal epithelium to initiate sensory transduction in olfactory sensory neurons. In this way the respiratory cycle controls temporal periods of odor stimulation, which enforce rhythmic activity throughout the bulbar neural circuit [1, 2]. Rhythmic patterns of activity can also be initiated in the absence of odor stimulation during inhalation when mechanoreceptors in olfactory sensory neurons are activated by air intake [3]. G protein-coupled receptors can further sustain this rhythmic activity [4], which propagates throughout cell populations [5–8] in the bulbar network and can be influenced by neuromodulation [9, 10]. The purpose of network oscillations driven by respiration in odor processing continues to be investigated, as respiration is hypothesized to generate a temporal window for odor processing [1, 5, 11–21].

Respiration can trigger inhibition in both the glomerular and granule cell layers to modulate second order mitral and tufted cells' (MTC) responses to odor stimuli [17, 22]. Individual MTCs connected to the same glomerulus are tuned by surrounding inhibitory networks to respond to inputs derived from respiration alone and/or odor stimulation during unique phases of the respiratory cycle [16, 18, 23]. It is still unclear why tuning across the respiratory cycle occurs and how it might facilitate odor processing. Furthermore, the separate and combined effects of respiration and sensory input responses in MTCs have not been fully characterized. During natural odor sampling, where odor presentation is coupled with inhalation, it is difficult to separate inhibition arising from odor input from that mediated solely by respiration. Here we employ a combination of *in vivo* optogenetic stimulation of sensory inputs and *in silico* modeling experiments, to investigate how inhibition that is activated by respiration can shape MTC processing of sensory input.

*In vivo*, we systematically stimulated discrete glomeruli at distinct temporal phases of the respiratory cycle using optogenetics. By recording the respiration cycle, the time of the optical stimulations, and the electrical activity of putative MTCs, we were able to explore how optically controlled spatiotemporal patterns of sensory input are processed during a respiratory cycle. We found that the precise interaction between sensory and respiratory activity was unique for individual mitral and tufted cells. We also found sensory inputs to be temporally gated across a respiratory cycle for neurons that also display spontaneous respiratory driven responses. Using a basic bulb model, we further investigated mechanisms that explain how respiration can activate periglomerular and granule cell inhibition to temporally gate sensory evoked responses.

## Materials and Methods

### Mice and MTC recordings

All the animals used in this study were treated according to the guidelines established by the U.S. National Institutes of Health, and the experimental protocols were approved by the Institutional Animal Care and Use Committee of the John B. Pierce Laboratory (JV1-2013). All wet lab experiments were performed in the John B. Pierce Laboratory, which is Association for Assessment and Accreditation of Laboratory Animal Care accredited. Electrophysiology was performed on mice expressing channelrhodopsin and yellow fluorescence protein (YFP) in their ORNs (OMP-ChR2, 30–100 days old). These OMP-ChR2 mice were generously provided to us by Dr. Venkatesh Murthy [18]. Mice were anesthetized with ketamine-xylazine anesthesia (100 and 20mg/kg i.p., respectively). Boosters were given at 25% of the original dose. Mice were maintained at physiological temperatures (36.5–38 degrees Celsius) while under anesthesia. A headcap was glued to the skull to enable stable positioning of the animal in the electrophysiological recording setup. A 3x3mm craniotomy was performed above a single olfactory bulb (OB) to expose the dorsal surface while keeping the dura intact. Saline was continually applied to the exposed outer dura to keep the tissue moist throughout the experiment. Quartz 5–10 MΩ micropipettes were advanced up to 400 μm into the OB neural layers at a 50-degree angle to target MTCs. Recordings up to this depth were deemed specific to the mitral cell layer. Post hoc histology was used to establish the *in vivo* recording method in one electrolytic lesion in each bulb of 3 mice. Extracellular spike recordings were sorted using a TDT RZ-5 system (Tucker Davis Technologies), which also stored all other experimental data time-synced with the neural data. The maximum firing of each neuron was calculated during periods of stimulation and intraburst firing. Since recording position alone cannot classify neuron types, only cells that produced maximum firing rates similar to typical mitral cells (<100 Hz) [24–26] were used in the optical stimulation experiments.

The respiration of the mouse was recorded continuously using a thermistor (Measurement Specialties, part G22K7MCD419, 30 ms response time) placed 3 mm from the nose. All mice were freely breathing throughout the entire experiment. Respiration periods were 671 ± 121 ms (average ± standard deviation) across 9 mice. One mouse displayed more variability in its respiration rate (1040 ± 309 ms, average ± standard deviation). Including or excluding this animal from the group data summary (polar plots and line graphs) had no influence of the significance of the figures, as its responses were very similar to the group average. At the end of each experiment animals were sacrificed with a lethal dose of pentobarbital sodium and phenytoin sodium (Euthasol).

### Optical stimulation

Optical stimulation of ORN axon terminals in the glomerular layer was performed using a 700mW 455nm laser fiber coupled to a custom-built digital micro-mirror device (DMD, Zinterscope, Guilford, CT). The DMD projector was connected via an HDMI cable and had a resolution of 1024x768 pixels (8-bit grey scale, 60 frames per second). Images are projected on an Olympus UPlanFLN 4x n.a. 0.13 objective, yielding an image size of 4x3 mm (4x4 μm projected pixel size) that was focused on the dorsal OB. The maximum power projected on the OB (laser at a 90% duty cycle and the entire DMD image turned on) was 100mW. This stimulation setup was designed around a modified Olympus Bx50WI microscope. Custom Matlab and Python scripts were used to control the DMD and the electrophysiology acquisition program from Tucker Davis Technologies, as well as to store and analyze data.

### Stimulation paradigms

Spatiotemporal coding of multiple dorsal glomerular inputs by a mitral cell was investigated using single light block stimulations that were presented one at a time onto the dorsal glomerular layer. The optical stimulus was comprised of a sequence of single blocks of light that were projected with a pseudo-random location on to the dorsal surface of the olfactory bulb (S1 Fig). By only presenting one light block stimulus at a time and by spatially spreading out the regions of stimulation, we prevented sequential stimulations to the same regions and greatly reduced the surface area of optical stimulation on to the bulb. Light blocks were small (25x25 pixels, 100x100 μm) and matched to the average size of a glomerulus (50–120 μm diameter [27]). These block stimulations were presented one at a time for 100 or 200 millisecond durations. Each stimulus was separated by an inter-stimulus interval of 200 milliseconds.

No stimulus repeatedly sampled the exact same glomerular region; rather the overlap of unique stimulus blocks was used to sample each dorsal glomerular region many times. By pseudo-randomly centering each block of light onto a unique location of the dorsal olfactory bulb, we did not bias our stimuli to a particular edge of a glomerulus or possibly activate two neighboring glomeruli repeatedly. We choose to not use a grid of stimulation blocks, but rather sample unique regions in a pseudo random order so that we could smoothly sample the edges of dorsal glomeruli. In contrast, a fixed stimulus grid is biased in that many stimulation blocks may miss the center of a given glomerulus and only target the edges of glomeruli, resulting in the possible mixture of primary excitatory responses and possible neighboring inhibitory responses. Stimulation with this paradigm lasted on average 35 minutes and included approximately 5,800 stimulations presented onto the dorsal olfactory bulb per single unit recording. Note that the same glomerulus was not stimulated repeatedly, rather a small block of light was used to sequentially sample all regions of the dorsal bulb independently. Heatmaps were generated from spikes recorded during each light block stimulus. Excitatory and inhibitory glomerular inputs were identified as the region of stimulation that activated or inhibited the recorded MTC the strongest.

### Data analysis: respiratory activity correction

The respiratory cycle was recorded using a thermistor. Respiratory phase angles corresponding to the onset of each random block stimulus were stored and used to generate temporally specific spatial response heatmaps. Determination of the respiratory phase angle (1 ms resolution) allowed for a precise characterization of respiration mediated phasic activity, which varied across the respiratory cycle. This method enabled each block stimulus to be sorted by its spatial position and/or the phase angle in which it was presented during the respiratory cycle. To isolate light stimulus evoked responses, the spontaneous respiration-evoked activity was subtracted. The average plus a minimum of one standard deviation of MTC activity during control (i.e, baseline, non-stimulated) periods was subtracted at each specific angle of respiration in which stimulation occurred. This resulted in the generation of heatmaps that display only spatially isolated stimulus-evoked responses (significantly greater than the average respiratory activity produced when no stimulus was present).

More specifically, all stimulus onset times were aligned to a phase in one half of the respiratory cycle (inhalation and exhalation), where each half's phase was separately and linearly scaled to time. The peak and trough of each respiratory cycle, as recorded by the thermistor, were defined as 0 (equivalent to 2π) and π radians, respectively. Recorded spikes were also assigned to a respiratory cycle angle, resulting in a vector of all recorded spikes relative to the peak and trough of respiration for each individual respiratory cycle. Angles were assigned to every millisecond of the longest recorded respiratory cycle, thus ensuring all angles for all recorded respiratory cycles were at a resolution of at least 1 ms. Several diagnostic simulator scripts were created that generated sets of test data that were subsequently analyzed to validate these analyses.

### Heatmaps

After K-means spike sorting (Tucker-Davis Technologies, OpenSorter), heatmaps of MTC spiking activity evoked by optical stimulation of all dorsal glomeruli were generated. Control respiratory activity associated with each response window was subtracted. Response windows began at, or shortly after, the onset of the light stimulus and terminated at least 50 ms before the end of the stimulation period. Control windows were selected to start at least 50 ms after light was turned off and end before the light was turned on.

Heatmaps displayed the stimulus-evoked MTC responses across the dorsal bulb. Evoked responses were shown by color-coding the stimulus blocks according to the number of spikes that the blocks evoked during the stimulus presentation, minus the number of spikes that occurred under control conditions during the same phases of the respiration cycle during which the block stimulation occurred. Heatmaps represented the average evoked response of all the random blocks of light presented in a trial, or a subset of blocks occurring within a specified range of phases within the respiratory cycle. Gaussian filters (sigma was set to 4 pixel units for excitatory regions of interest and 10 for inhibitory regions of interest) were used when generating heatmap images.

From these heatmaps, primary sites of excitation and inhibition were identified. Excitatory regions of interest (ROI) were identified as sites that significantly excited the recorded neuron to levels above average respiratory activity plus multiple standard deviations (described in the results). The respiration-driven activity associated with the respiratory cycle angles of each stimulation window was subtracted to isolate discrete excitatory or inhibitory dorsal glomerular regions. Responses to all stimulation blocks intersecting those excitatory ROIs were isolated and used to generate polar plots of MTC activity to stimulation at glomerular sites across twelve phases of the respiratory cycle. These polar plots were generated to examine how stimulation of single glomerular inputs can gate mitral/tufted cell activity across various phases of the respiratory cycle.

Inhibitory ROIs were also identified by generating heatmaps where the respiratory activity was not subtracted. This allowed us to isolate sites of dorsal glomeruli that reduced MTC activity below average control levels of respiratory activity. These ROIs were isolated as dark regions in the heatmap as they were associated with sites of stimulation that reduced baseline levels of MTC activity.

### Directional statistics

Polar plots were used to examine changes in MTC activity across the respiratory cycle. Stimulations were sorted by the phase angle they occurred at relative to the respiratory cycle. Evoked and control responses corresponding to specific phases of respiration were compared. Directional statistics (Matlab Circular Statistics Toolbox written by Philipp Berens in 2006) confirmed respiratory phasic activity in each recording (Rayleigh’s test for non-uniformity) and allowed us to identify significant shifts in MTC activity following optical stimulation (non parametric multi-sample test for equal medians, similar to a Kruskal-Wallis test for linear data). Changes in MTC centroid angle (radians) were examined. A centroid is the vector mean of all data points in the plot under a given condition. Group statistics, mean, standard deviations (SD), and standard error of the mean (SE) were compared. Two-tailed unpaired t-tests (across simulated and control groups) were used to compare changes in centroid angle, and peak and average firing rates. Peristimulus histograms were plotted in time and aligned by the onset of the stimulus (time = 0 sec). Stimulations were grouped by their onset times relative to the respiration cycle.

#### Diagnostic model

To verify the data analysis code, artificial data were created with known statistical properties. We used periodic Gaussian stimulus event probability distributions whose peaks aligned with defined respiration cycle phase angles. These data sets were created with Matlab scripts we made available in ModelDB (accession number 183300). They were used to verify the experimental analysis code for each set of electrophysiological and computationally modeled data. This diagnostic data confirmed that the analysis code returned the correct results from data with known properties.

### Computational modeling

The role of respiration in mediating inhibition and sensory input was investigated using a model containing two glomeruli that received both respiratory-driven activity and sensory inputs. These two inputs were used to mimic our optical block stimulus in our *in vivo* experiments. Respiratory activity included a burst of action potentials that was synchronized to the respiratory cycle's transition from inhalation to exhalation. Respiration mediated burst firing was set to occur at 2.5 Hz in all simulations unless otherwise specified in the results. As in the electrophysiological experiments, recordings were made from a single cell, here modeled as a mitral cell. The mitral cell (MC) received either periglomerular (PG) inhibition, granule cell (GC) inhibition, both PG and GC (PG+GC) inhibition, or no lateral inhibition. All possible network combinations were explored. The design of the model was guided by the microcircuit connectivity information of glomerular-mitral cells columns and associated periglomerular and granule cell lateral inhibitory synaptic connections: see Fig 5.5 in [28]. The model was written in NEURON 7.4 [29] and incorporated previously published models of mitral, periglomerular, and granule cells from selected network model codes available in ModelDB [30]. Our periglomerular inhibition incorporates both inter- and intra-glomerular connections and therefore our findings from this model may resemble activities in a myriad of subtypes of periglomerular cells and short axon cells [31]. The mitral cell and granule cell models were from ModelDB, accession number 144054 [32], and the periglomerular cell was from ModelDB, accession number 149739 [33]. Respiratory mediated events were present on each column (mitral cell tuft), while the simulated input mimicking the light stimulus in our *in vivo* experiments was applied to either the parent glomerulus (the same MC that was recorded, **primary glomerulus input model**), or an adjacent column's mitral cell (**secondary glomerulus input model**).

A second model, the **glomeruli connectivity model**, further investigated how glomerular inhibition can shape respiratory gating of sensory inputs by varying the number of connected glomeruli and associated mitral and inhibitory interneurons (glomerular columns). Basic glomerular columns were modeled in the same fashion as the **primary and secondary glomerulus input models**: with one mitral cell and one or two types of lateral inhibitory cells. Zero to 90 connected glomerular columns were explored, although only up to 6 columns are shown in the results as trends in modeled mitral cell activity plateaued. Polar plot analyses of response gating during a single respiration were generated and compared with experimental data. The model code is available on ModelDB, accession number 183300. We used the Louise cluster at the Center for High Performance Computation in Biology and Biomedicine at Yale University and the Neuroscience Gateway (NSG) [34].

We used the excitatory and inhibitory synapses from [32] for the AMPA-NMDA and GABA-A synapses representing the olfactory nerve excitatory synapses and the reciprocal and inhibitory synapses between the cells. We used the default synaptic strength given in [32] for all excitatory and inhibitory synapses and kept it constant in each stimulation (no synaptic plasticity). For the granule cells we used higher levels of inhibition, specifically 4 times greater than the periglomerular cells unless otherwise stated in the results. Up to 90 times higher granule synaptic inputs as compared to periglomerular synaptic inputs were investigated to explore the onset and upper range of granule cell effects, as their inhibitory effects were weaker than those observed in response to periglomerular inhibition.

Although not the main focus of this paper, we also investigated the possibility that external tufted cells (ETCs) may affect the gating of sensory input evoked MTC excitation. Excitatory synapses (AMPA-NMDA) were created between external tufted cells and periglomerular neurons and between external tufted cells and the apical tuft of the mitral cells. All other synaptic connections remained the same. The ETC was created by halving the size of the mitral cell model and removing the secondary dendrites.

We systematically varied the intensity of respiration versus stimulus-evoked inputs. We used a Gaussian Poisson rate with a half width of 30 ms to model the occurrence of excitatory events for either respiration or light driven stimulus. The Gaussian light stimulus events were convenient to study the relative strengths of respiration versus light stimulation. Combinations of R (respiration Gaussian peak Poisson rates) and S (sensory Gaussian peak Poisson rates) were used in these simulations: R and S were varied from 20 to 760 Hz in steps of 20 Hz (1444 combinations). A table that relates the R and S peak of the Gaussian Poisson rates to average numbers of excitatory events per respiratory cycle and event rates (Hz) is provided in S2 Fig. When multiplied by the 4 network types (PG, GC, PG+GC and no inhibition) and 2 stimulation choices (primary versus secondary glomerulus inputs), this resulted in 11552 model runs. An additional 5776 simulations tested higher levels of GC inhibition (30, 50, 70, and 90 times greater than the PG input). Additional simulation with R and S values within those ranges were also tested (R from 300 to 495 Hz in steps of 5 Hz were all paired with S from 50 to 195 Hz in steps of 5 Hz for a total of 1200 simulations). A version of the secondary glomerulus model with inhibition by secondary mitral cells was also examined (2888 simulations).

## Results

**Fig 1:**
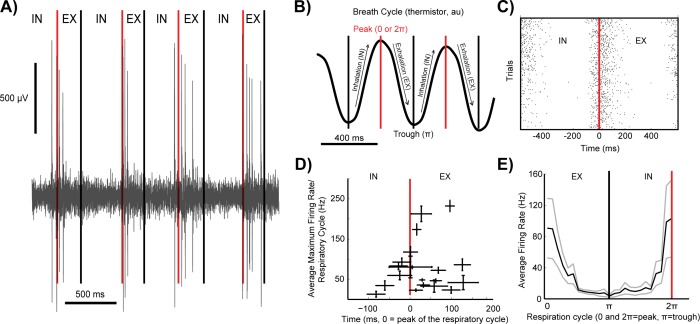
MTCs exhibit burst firing at the transition from inhalation to exhalation. (A) Spontaneous burst firing in an *in vivo* extracellular single unit recording from the mitral cell layer. (B) A thermistor recording of respiratory cycle activity. Black arrows denote periods of inhalation and exhalation. Red and black lines highlight the peaks and troughs, respectively, of each thermistor respiratory cycle. (C) A raster plot of spikes across the respiratory cycle of the neuron in panel A. Red line at 0 ms is the time of the peak of the respiratory cycle. (D) Average maximum firing rates (Hz) relative to the thermistor peak voltage (n = 21 single unit recordings, 20 mice) and (E) across the respiratory cycle (cycle angles 0 to π to 2π, radians) shown in black, plus and minus standard errors in grey (n = 10 single unit recordings, 8 mice).

All MTCs investigated in this study exhibited oscillatory burst firing (see example voltage trace, Fig 1A) following the transition from inhalation to exhalation, as recorded with a thermistor (Fig 1B). Each MTC passed the Rayleigh’s test for non-uniformity (p < 0.05, Matlab Circular Statistics toolbox), meaning the neurons display significant coupling of their baseline activity with respiration. Alignment of the peak and trough of the thermistor recordings were used to define the transitions between exhalation and inhalation (red and black lines in Fig 1A–1E). The maximum instantaneous burst firing rates and their timing relative to the transition from inhalation to exhalation was unique for each recorded neuron (Fig 1D and 1E). Of all neurons recorded in the mitral cell layer, 10 cells produced intraburst maximum firing rates similar to typical mitral cells (<100 Hz) [24–26] and were further examined using optical stimulation of olfactory sensory neurons expressing channelrhodopsin.

### Respiration gates stimulus evoked excitation

**Fig 2:**
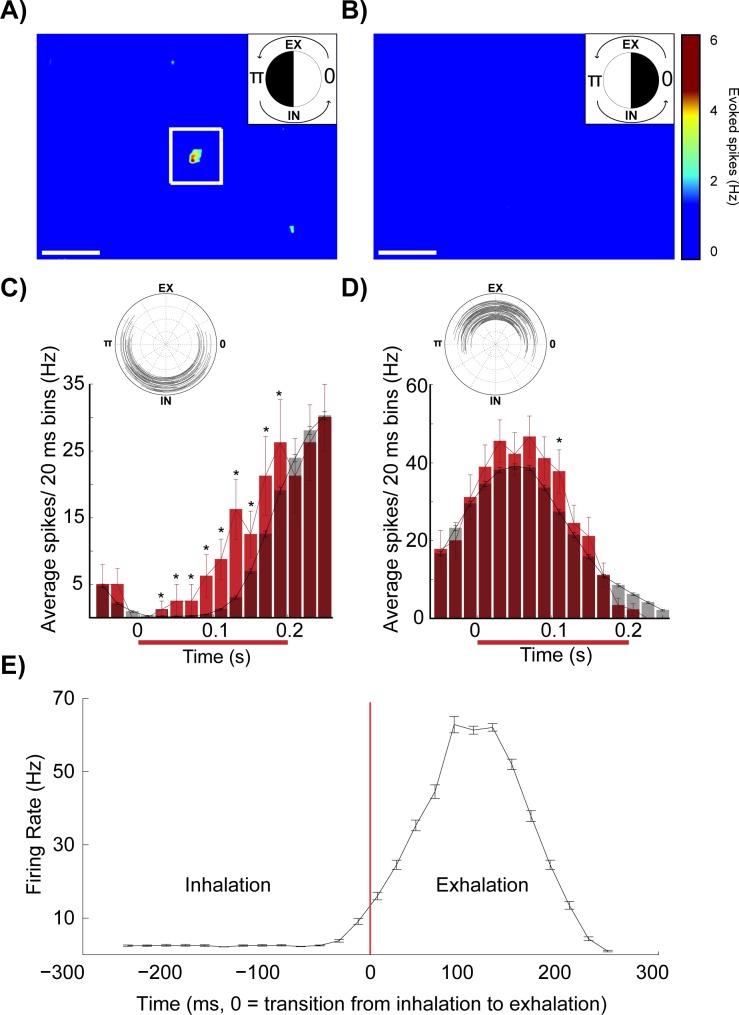
Isolation of excitatory glomerular inputs in neurons with strong and regular respiratory activity. Composite heatmaps of averaged evoked spikes evoked by overlapping light blocks presented to dorsal glomeruli (7,213 stimulations). Stimulations were grouped by their start times that fell within specific ranges of the respiratory cycle indicated by the upper right circle plots in each panel. Black coloring indicates the phase from which stimulation blocks were selected to generate the heatmap. (A) Stimulation onsets occurring during π +/- π/2 radians (3632 stimulations) and (B) occurring during 0 +/- π/2 radians (3,581 stimulations) are shown in these heatmaps with the average plus 1.5 SD of respiratory activity subtracted. White scale bars in panels A and B are 1 mm. (C) Peristimulus response histogram for stimulation onsets presented during the transition from exhalation to inhalation that hit the excitatory region of interest (white box in A). Grey bars represent control baseline activity when no stimulus was present. Red bars represent stimulated evoked responses. Inset represents stimulation periods (black lines, 36 stimulations, starting during 7π/6 +/- 5π/12 radians). This was the phase in which this MTC neuron responded best to primary excitatory glomerular input (ROI in panel B). (D) Similar to the peristimulus histogram presented in C, but stimulation onsets occurred during the transition from inhalation to exhalation (46 stimulations starting during π/6 +/- π/3 radians). All stimulation onset times were aligned to 0 on the x-axis in panels C and D. (E) The respiratory activity that was subtracted in panels A-D. Error bars equal to the standard error. *: P < 0.05. All firing rates were aligned to the transition from inhalation to exhalation (red bar, 0 ms). The x-axis in panels C-E is in real time. Stimulation onset is aligned to 0 in C and D; the transition from inhalation to exhalation is aligned to 0 in E.

**Fig 3:**
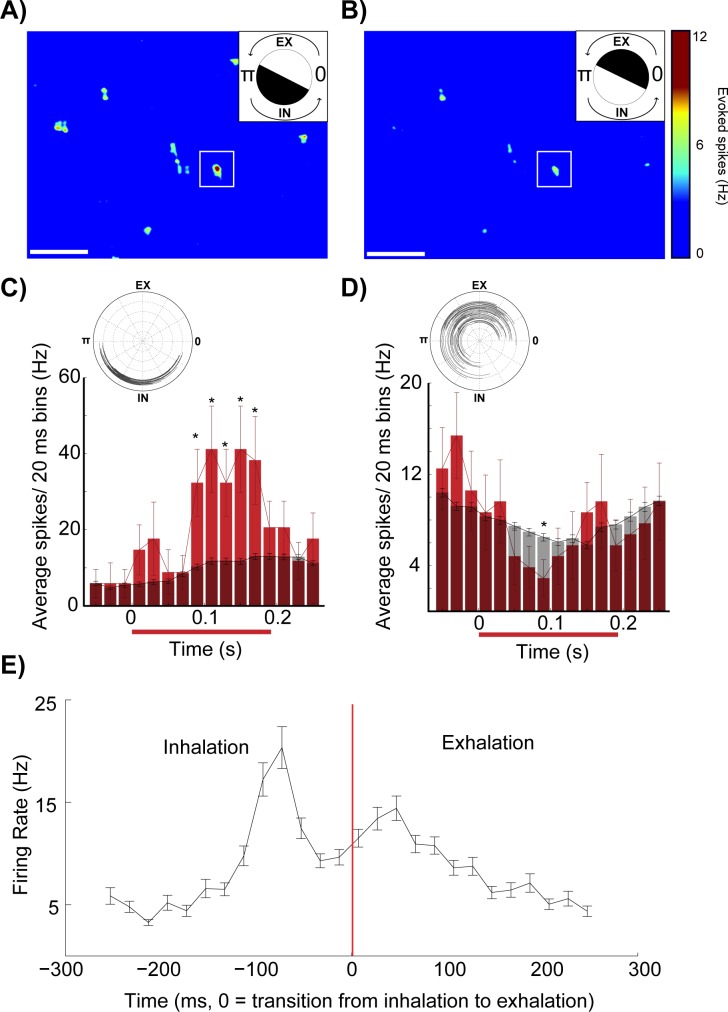
Isolation of excitatory glomerular inputs in neurons with weak, irregular respiratory activity. Similar composite heatmaps as in Fig 2 of averaged evoked spikes recorded from a single MTC (8,512 stimulations). (A) The average respiratory activity plus 10 standard deviations of respiratory activity was subtracted. Only stimuli presented during the specified range of the respiratory cycle angles (early inhalation, stimulations start during 7.5π/6 +/- π/2 radians) were used to generate this heatmap. (B) Same as A, but only response-weighted blocks from stimuli presented during early exhalation are displayed (stimulations start during 1.5π/6 +/- π/2 radians). White scale bars in panels A and B are 1 mm. (C) Peristimulus histogram of the response profile to stimulation (red) of the excitatory region of interest (white box in A and B) with onsets during inhalation. Control respiratory activity in the absence of optical stimulation is shown in grey. Inset represents periods (black lines) associated with stimulations with onsets during the early inhalation phase (23 stimulations started during 7π/6 +/- π/6 radians). This was the phase in which this MTC neuron responded best to primary excitatory glomerular input (ROI in panel A). (D) Similar to the peristimulus histogram presented in C, but stimulation onsets shown in this panel occurred during the exhalation phase (41 stimulations start during 11π/6 +/- π/2 phase angles). All stimulation onset times are aligned to 0 on the x-axis in panels C and D. (E) The respiratory activity that was subtracted in panels A and B. Error bars equal to the standard error. *: P < 0.05. All firing rates were aligned to the transition from exhalation to inhalation (red bar, 0 ms). The x-axis in panels C-E is in real time.

It was necessary to account for the natural variation in the frequency and periodicity of respiratory cycle activity (Fig 1E) in order to isolate excitatory glomerular inputs. Stimulations were sorted by their onset times relative to the respiratory cycle in order to compare pre and post stimulus time periods during similar phases of the respiratory cycle (Figs 2E and 3E). The average phase-specific respiratory activity plus 1 or more SDs associated with each unique stimulus period was subtracted (Figs 2A, 2B, 3A and 3B) to isolate primary sites of glomerular evoked excitation. Each neuron showed a preference for excitation by our optical stimulus during a specific phase of the respiratory cycle (Fig 2A and 2B). By characterizing the separate effects of respiration (control periods of no stimulation) and MTCs evoked responses to light stimulus activation of sensory inputs, putative excitatory inputs were isolated using our panel of pseudo-randomly placed single light block stimuli. These data indicate that the timing of glomerular stimulation during a respiration influences the efficacy of evoked excitation in MTCs.

The majority of MTCs displayed very sharp coupling of baseline activity to the respiratory cycle under control conditions. This allowed for a clean isolation of hot spots of excitatory glomerular input following the subtraction of respiratory activity (Fig 2A). Evoked responses associated with stimulation at hot spot sites in the heatmaps were only observed when stimulation occurred during preferred phases of the respiratory cycle (Fig 2A) and not during other phases (Fig 2B). In this example neuron, an excitatory glomerular region was isolated following subtraction of the average respiratory activity plus 1.5 SD, which ranged from 2 to 53 spikes across exhalation and inhalation phases, respectively (Fig 2E). Stimulation of the putative excitatory glomerulus during the transition from exhalation to inhalation evoked activity of the recorded MTC (π radians, Fig 2A and 2C). Stimulation of the same site during the early exhalation phases did not evoke an increase in neuron spike rates (Fig 2B and 2D).

Strong coupling with respiration was associated with a prominent gating of light stimulus evoked excitation to a specific phase of the respiratory cycle (example neuron’s respiratory activity Fig 2E, n = 7 neurons). This group of neurons had an average respiratory activity of 18.2Hz ± 22.5 SD, ranging from as low as 4.3 Hz to as high as 68 Hz across each respiratory cycle. These neurons required control activity plus 0–3 SD to be subtracted before excitatory hot spots were clearly isolated.

In contrast, three MTCs displayed more variability, yet still showed significant coupling of baseline activity with respiration (Rayleigh’s test for non-uniformity, p < 0.05). Variable phasic activity resulted in somewhat noisier heatmaps (an example is shown in Fig 3A and 3B). However, after subtraction of the average respiratory activity, excitatory regions of interest were visible. The threshold for evoked activity was increased by subtracting a conservative 10 additional SD of respiratory activity to isolate putative significant excitatory glomerular inputs (15–23 spikes above control levels of respiratory mediated MTC activity, Fig 3C–3E). Although these neurons were excited by the optical stimulation during a large part of the respiratory cycle (Fig 3A and 3B), the cells did show gating of stronger evoked responses to specific phases of inhalation (Fig 3C, inset shows cycle angles 3.66 +/- 1.57 radians in black, which corresponds to the period of stimulation used to generate the heat map). Significant evoked responses were readily identified in the peristimulus histograms (Fig 3C). Although not consistent, some stimulations that occurred during exhalation resulted in a slight reduction in firing rates below baseline levels associated with respiration (Fig 3D). These three neurons displayed less regular, yet still significant coupling to respiration. Although they were excited during a large part of the respiratory cycle, stimulus evoked responses still occurred predominantly during the inhalation phases. These three neurons had a wide range of average respiratory activity (0.5 Hz, 8.7 Hz (example neuron in Fig 3), and 86.8 Hz).

**Fig 4:**
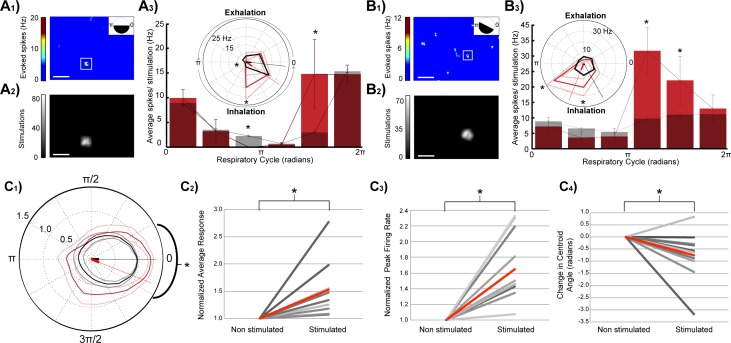
Phasic respiratory firing gates sensory input evoked responses. (A_1_) Stimulus evoked response heatmaps corresponding to sites of light block stimulation of dorsal glomeruli layer. Upper right inset indicates phases of the respiratory cycle (black coloring) in which stimulation blocks were selected to generate the heatmap. (A_2_) Random blocks of light presented during all phases of the respiratory cycle that hit the excitatory region of interest were isolated and (A_3_) used to generate a bar graph and corresponding polar plot (inset) showing how evoked spike rates (Hz) from the same stimulation site (shown in A) vary when stimulations were triggered during 6 ranges of the respiratory cycle (85 stimulations total; 13, 7, 18, 15, 12, 20 stimulations per 1/6^th^ cycle angle range, 878–1076 control measurements per 1/6^th^ cycle angle range). Stimulation onsets that began within each 1/6^th^ of the cycle were grouped together and the post stimulus period for each stimulation was analyzed. Corresponding control windows also began within each 1/6^th^ of the respiratory cycle. Red/pink = stimulated, black/grey = control. SE is displayed as error bars in the bar graphs and grey or pink lines in polar plots. (B_1-3_) Same as panel (A_1-3_) but a different cell (77 stimulations total; 15, 10, 11, 14, 11, 16 per 1/6^th^ cycle angle range, 1173–1234 control measurements per 1/6^th^ cycle angle range). White scale bars in panels A_1_, A_2_, B_1_, B_2_ are 1 mm. (C_1_) Average group polar plot (10 neurons) aligned by the control centroids which were all rotated to respiratory cycle polar angle 0 and scaled to control peak = 1. Stimulated polar plots were rotated and scaled by the same amounts as their associated control plots. Plots from the two groups (control = black and stimulated = red) were then averaged and had their SEs calculated and graphed in lighter colors. (C_2-3_) Summaries of normalized changes MTC average response (evoked firing) and peak firing across the respiratory cycle. Data was normalized to control measurements by dividing the average firing rate and peak firing rates without stimulation. The average normalized change under stimulated and control conditions (red line). (C_4_) Change in centroid angle of control and stimulated recordings. The average change (red line). (10 neurons, 3542–8511 stimulations, an average of 5869 stimulations per neuron). *: P < 0.05.

The strength of MTC excitation depended on the phase of respiration at which the stimulus occurred (white box, Fig 4A_1_, superimposed stimulation blocks, 85 trials, Fig 4A_2_). MTC activity increased significantly when the stimulus onset occurred during phase angles that *preceded* the respiratory evoked burst firing (Fig 4A_3_). Conversely, when this excitatory region of interest was optically stimulated during periods immediately *following* phasic respiratory firing, no excitation of the recorded MTC was observed and sometimes a slight reduction in activity below baseline respiratory activity was recorded (Fig 4A_3_). Relatively weaker coupling with respiration also resulted in preference for excitation during the inhalation phase (Fig 4B_1-3_, 77 stimulations, same neuron in Fig 3). Across neurons, significant stimulus evoked increases in activity were observed immediately before and during periods of respiratory evoked activity (all polar data was shifted relative to peak control burst firing, which was aligned to t = 0; activity was normalized to peak control firing rates for each neuron, Fig 4C_1_, 9 animals, n = 10 neurons, * = P<0.05). Simple t-tests were used to compare activity across each range of respiratory phase angles. Directional statistics confirmed significant shifts in centroids (P<0.0001) across stimulated and control groups (non parametric multiple-sample test for equal means, similar to Kruskal-Wallis test for linear data, Matlab Circular Statistics toolbox).

Group data indicate that optical stimulations of isolated excitatory dorsal glomeruli across the complete respiratory cycle resulted in significant increases in normalized average responses (Fig 4C_2_, P < 0.005, stimulated = 26.0 ± 10.1 Hz (mean ± SE), control = 21.5 ± 9.1 Hz (mean ± SE)), polar areas (not shown, P = 0.021, stimulated = 5131.9 ± 3375.4 Hz^2^ (mean ± SE), control = 4034.6 ± 2655.0 Hz^2^ (mean ± SE)), and peak firing rates (Fig 4C_3_, P < 0.001, stimulated = 67.8 ± 32.5 Hz (mean ± SE), control = 50.4 ± 25.7 Hz (mean ± SE)). Interestingly, across all neurons, there was a significant shift in centroid angle following stimulation of excitatory glomeruli. This shift in activity was towards earlier phases of the respiration cycle (Fig 4C_4_, P = 0.036, stimulated = 5.3 ± 0.6 radians (mean ± SE), control = 6.0 ± 0.3 radians (mean ± SE)). Gating was measured as a change in the centroid angle (radians) across control and stimulated groups.

Each MTC displayed a unique window for sensory input processing, although as a group, excitation of MTCs by dorsal glomeruli was consistently observed during late exhalation and early inhalation periods of the respiratory cycle. No stimulus-evoked excitation was observed following periods of respiration-mediated burst firing (Fig 4C_1_). This strength of coupling with respiration was associated with more narrow phases of the respiratory cycle during which optical stimulation excited the recorded MTC. These results suggest that respiration functions as a gate for excitatory sensory inputs.

### Inhibition of MTC activity

**Fig 5:**
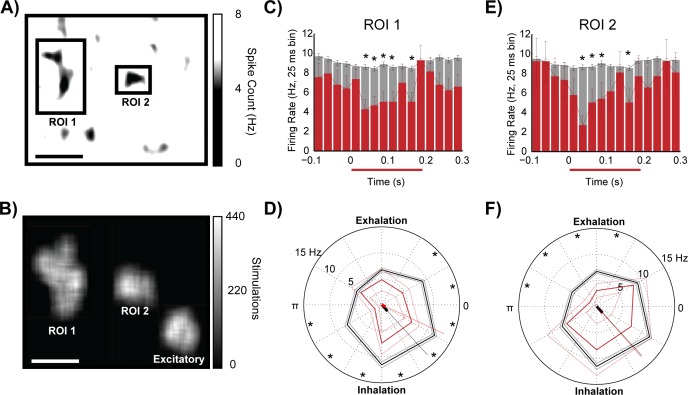
Mapping of inhibitory regions. (A) Spikes associated with each random block of stimulation were averaged across the stimulation space on the dorsal glomerular layer. Respiratory activity was not subtracted. Activity in the heat map was thresholded at 5 Hz to highlight regions of little activity in response to random block stimulation. Sites of inhibition were isolated (ROI 1 and 2) as regions of black where low MTC activity was observed when light stimulation excited those glomerular areas. Black scale bar is set to 1 mm. (B) Random blocks of stimulation hitting the two selected regions of interest (ROI). White scale bar is set to 1 mm. Data from single unit recordings in response to optical stimulation of glomeruli in ROI 1 (C,D) and ROI 2 (E,F). (C,E) Peristimulus histograms of the average responses to stimulating ROI 1 (104 stimulations) and ROI 2 (211 stimulations). Stimulus onset times are aligned at time 0s. Stimulations presented during all phases of the respiratory cycle are displayed. Stimulated ± SE (red) and control ± SE (grey) polar plots showing spike rates (radii) post stimulation (red) and no stimulation (black) at (D) ROI 1 and (F) ROI 2. ROI 1 was stimulated 104 times (13, 19, 24, 22, 8, 18 per 1/6^th^ cycle angle range) and had 1168–1230 control measurements per 1/6^th^ cycle angle range. ROI 2 was stimulated 220 times (42, 36, 39, 31, 30, 42 per 1/6^th^ cycle angle range) and had 1168–1230 control measurements per 1/6^th^ cycle angle range. *: P < 0.05.

Glomeruli that inhibited MTC activity to levels below that evoked by respiration alone were also isolated using the random block stimulation paradigm. Heatmaps were created with a threshold of 8 Hz, which was the average spontaneous activity of the recorded neurons under control conditions. Heatmaps highlight regions of dorsal glomeruli that evoked a reduction in MTC activity that was significantly below respiratory levels (black areas, Fig 5A). Inhibitory regions of interest were isolated and stimulation blocks activating those inhibitory sites were plotted (ROIs 1 and 2, Fig 5B) and compared with the site of excitation (previously identified in Fig 3D and labeled ‘excitatory’ in Fig 5B).

The average peristimulus response profiles of optical stimulation of inhibitory dorsal glomeruli (ROI 1 Fig 5C and ROI 2 Fig 5E) across all phases of the respiratory cycle resulted in a net inhibition that lasted throughout most of the stimulus window. This inhibition was subtle, (approximately 2 spikes per 25ms time bin) and could only be isolated after accounting for baseline respiratory activity. The strongest response to stimulating the excitatory ROI occurred at 80–160 ms following stimulation of the excitatory ROI (Fig 3F). This same period was examined to see if activation of the inhibitory ROI 1 could inhibit MTC activity. Light activation of ROI 1 resulted in inhibition of the MTC to levels below respiration levels during the inhalation phase and at the beginning of the exhalation phase (Fig 5D). Stimulation of a second site of inhibition (ROI 2) also resulted in inhibition of the same MTC neuron, but this occurred during the exhalation phase (Fig 5F).

**Fig 6:**
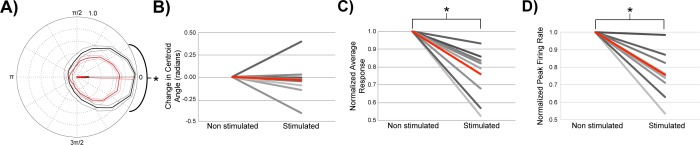
Stimulation of inhibitory glomeruli reduces respiratory activity. (A) Across cells, inhibition of baseline spontaneous and respiratory activity by optically stimulated glomeruli was observed irrespective of when stimulation occurred during the respiratory cycle (8 animals, 10 cells, 11 inhibitory glomerular inputs). Average group polar plot aligned by the control centroids which were all rotated to respiratory cycle polar angle 0 and scaled to control peak = 1 (control = black and stimulated = red, SE graphed in lighter colors, similar to Fig 4C_1_). (B) The centroid angle of MTC activity shifted in response to stimulation of most inhibitory glomerular regions, but this shift was not in a consistent direction. (C) There was a significant decrease in the average MTC firing rate across the respiratory cycle following stimulation of inhibitory dorsal glomeruli. All data were normalized to the average polar radius of the control, non-stimulated measurements. (D) The majority of MTCs displayed a significant decline in peak firing rates, when normalized to control levels. *: P < 0.05.

Across neurons, optical stimulation of putative inhibitory glomeruli resulted in an inhibition of respiration evoked MTC burst firing (Fig 6A, *: P < 0.05). Neurons did not display significant shifts in activity (centroid angle) toward earlier or later phases of the respiratory cycle in response to optical stimulation of dorsal glomerular regions of inhibition (Fig 6B, stimulated = 0.114 ± 0.50 radians (mean ± SE), control = 0.108 ± 0.57 radians (mean ± SE)). Confirming the isolation of glomeruli that inhibit MTC activity, MTCs displayed significant reduction in polar radius (Fig 6C, P < 0.0001, stimulated = 28.5 ± 8.6 Hz (mean ± SE), control = 33.7 ± 10.1 Hz (mean ± SE)) and polar area (p<0.0001, stimulation = 4744.8 ± 2322.9 Hz^2^ (mean ± SE), control = 6926.3 ± 3378.2 Hz^2^ (mean ± SE)), as well as a decline in peak firing rates following stimulation of inhibitory regions of interest (Fig 6D, p<0.0001, stimulated = 71.8 ± 27.4 Hz (mean ± SE), average control = 106.2 ± 32.0 Hz (mean ± SE), for 9 of the 11 regions of inhibition, 10 neurons, 9 animals).

Our *in vivo* experiments revealed a temporal gating of excitatory sensory input to periods before and during phasic respiratory activity, whereas inhibition of MTCs by sensory input was not temporally gated across the respiratory cycle. Reductions of MTC activity following stimulation of glomeruli, which activated putative inhibitory inputs, were found to occur across all phases of respiration.

### Computational modeling of respiration evoked inhibition

**Fig 7:**
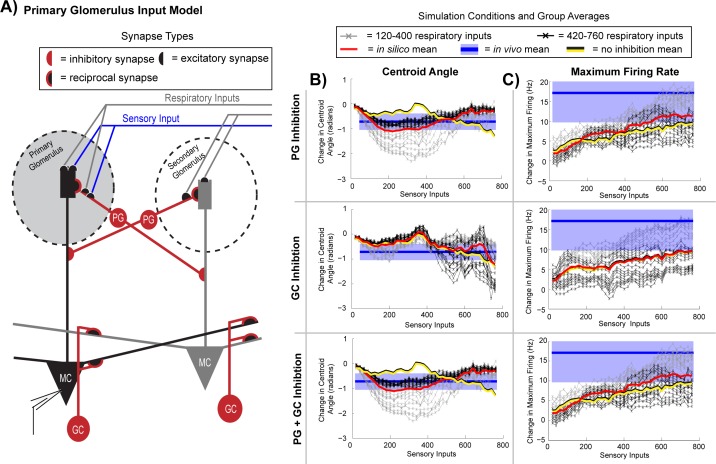
Periglomerular inhibition driven by respiration gates mitral cell activity across the respiratory cycle. (A) In our circuit model, one mitral cell (black) receives excitatory sensory inputs (blue line) at its glomerulus (large grey circle). Inputs driven by respiration (grey lines, entering two separate glomeruli) have excitatory synapses on the apical tuft of two separate mitral cells. (B) Changes in the centroid angle comparing sensory evoked and respiratory mitral cell activity were examined across varying levels of sensory (x-axis) and respiratory input (lines); results are from simulations that contained only periglomerular neurons (upper row), only granule cells (middle row), and both granule and periglomerular neurons (bottom row). (C) Comparisons of maximum firing rates across simulations types, as in (B). Color key depicting the strength of the respiratory inputs (120–760 synaptic inputs): thick red line = average activity across varying respiratory input strengths (120–760 synaptic inputs), black/yellow line = average activity without lateral inhibition, blue line = the *in vivo* mean and blue shaded region *in vivo* mean ± standard error of the mean (SE) recorded across 10 neurons).

Gating of sensory inputs by respiratory activity and respiration driven lateral inhibition was further investigated using computational models. With the model we explored two different paradigms of sensory input and how they influence mitral cell activity: (1) primary glomerular input, which provided inputs to the mitral cell recorded in the simulation (Fig 7A) and (2) secondary glomerular inputs, which provided inputs to neighboring mitral cells (S8 Fig). Respiration was simulated to evoke excitation in all glomeruli. Inhibitory synaptic connections between the two mitral cells were made by periglomerular and/or granule cell synapses. The relative strengths of respiratory and sensory inputs to the glomeruli were systematically varied to find if specific ratios of *respiration* to *sensory driven* inputs replicated the gating of evoked MTC activity observed *in vivo*. These simulations used a basic neuron model of a mitral cell, which likely represents the majority of neurons recorded *in vivo* as their intraburst peak firing rates were below those typical of tufted cells [24–26]. Further investigation of the differences in how respiration modulates sensory processing in identified tufted and mitral cell populations is prudent. With our simulations we also investigated the independent and combined roles of inhibitory periglomerular (PG) or granule (GC) cells by systematically activating either or both.

Periglomerular neurons were the main drivers of shifts in centroid angle that occurred after stimulation of a sensory input (Fig 7B, upper panel, averages with periglomerular inhibition (red), without inhibition (yellow/black), *in vivo* data (blue)). In contrast, high levels of granule cell inhibition (30 times greater than that of the PG neuron) only weakly shifted sensory evoked mitral cell activity (centroid angle) across the respiratory cycle. These shifts were similar to stimulations without lateral inhibition (Fig 7B, middle panel). In the simulation with only GC inhibition, less pronounced shifts in the centroid angle to earlier phases of the respiratory cycle were observed across all respiratory input strengths when the sensory input was comprised of up to 400 excitatory events. An exception to this occurred at very high sensory and respiratory drive, which may be not be physiologically relevant as this did not increase the maximum firing rate (black lines, sensory inputs > 400, Fig 7C). When both granule cell and periglomerular cell inhibition were present, minimal interactions between the two drive levels were observed, as periglomerular mediated responses overrode those mediated by granule cells.

Even without lateral inhibition, relatively weaker shifts occurred in the phase of mitral cell activity, especially at higher levels of sensory drive, which increased firing rates approaching the *in vivo* results (Fig 7B, compare no inhibition simulation data average (black/yellow line) to average *in vivo* recordings (blue)). A refractory period followed phasic respiratory activity and prevented the mitral cell from responding to additional sensory inputs. During periods outside of this refractory window, sensory evoked mitral cell activity shifted to earlier periods of the respiratory cycle (see S3 Fig for detailed comparison of neuron activity of the different cells in the model).

The average shift in centroid angle observed *in vivo* was -0.75 radians (blue line, ~1/8 revolution, Fig 7B). In our simulation, at lower sensory input strengths, all respiratory inputs were capable of achieving a 0.75 radian shift in centroid angle between sensory input stimulated and control conditions. As the strength of the sensory input increased, lower respiratory input strengths evoked stronger shifts in mitral cell activity. In the models with periglomerular inhibition, these shifts were greater than those observed *in vivo*. In models with only granule cell inhibition, higher respiratory input strengths evoked the strongest shifts in mitral cell activity when the sensory input was strong (> 400 excitatory events), and only with strong sensory input were these shifts in mitral cell activity greater than what was observed *in vivo*.

**Fig 8:**
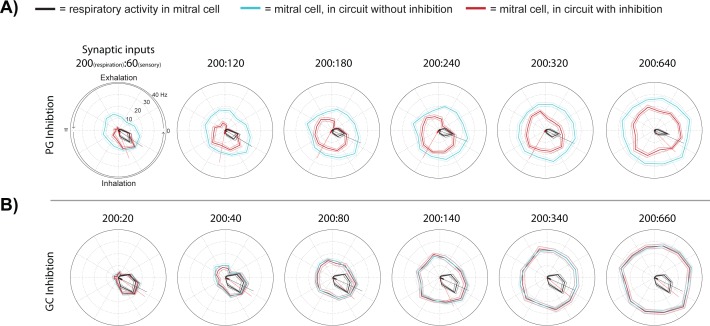
Gating of evoked responses in mitral cell activity is influenced by the ratio of simulated sensory to respiratory input strengths. Polar plots of stimulated (red, pink: ±SE), control (black, grey: ±SE) sensory input conditions, and a positive control with sensory and respiratory inputs in the absence lateral inhibition (blue, light blue: ±SD). Respiratory inputs for each plot are set to 200 and stimulation inputs are varied from 60 to 640, as shown in the ratio above each plot (respiratory input: stimulus input). Radii (y-axis) scale and respiratory cycle angles (radians) shown in the upper left polar plot is the same for all polar plots. (A) Simulations containing only PG inhibition present and (B) simulation containing only granule cell inhibition. *: P < 0.05.

Statistics similar to those performed on our *in vivo* datasets were used to evaluate how excitation of the primary glomerular input influenced gating of MTC excitation across the respiratory cycle (Fig 8). Strong phase gating in simulated sensory evoked and respiratory activity by PG neurons was present (Figs 8A and S4). At relatively lower sensory input strengths (60–240 synaptic inputs) gating of evoked mitral cell activity was observed when the respiratory input were set to 200 (cf. Fig 7). As the strength of the excitatory inputs increased and became stronger than the respiratory input, the gating effect was lost (<80 excitatory sensory inputs events, see Fig 7E). The limited role of GCs in respiration mediated lateral inhibition gating across the respiratory cycle was supported by nearly identical responses across circuit models where no inhibition (polar plots shown in blue) or GC inhibition was present (Fig 8B).

These simulations indicate that PGs temporally gated MTC sensory activity. The specific mechanism by which PG neurons gate sensory inputs were further explored in our simulations. By systematically removing either the intraglomerular PG reciprocal synapse or the lateral interglomerular inhibitory synapse (S5 Fig), we isolated the intraglomerular inhibitory synapse as being primarily responsible for directing the gating of sensory evoked responses. That is not to say that the interglomerular lateral inhibitory synapse may not also be important for phase gating *in vivo*, but in our simulations here it did not appear to play a prominent role in sensory input gating. Further investigation of how respiration may direct interglomerular inhibition and specifically the role short axon cells may have in modulating PG and external tufted cell activities [35] may aid in understanding their roles in temporal gating of sensory input during each respiratory cycle.

We began to explore the effects of adding intraglomerular external tufted cells (ETC) synapses to the model (S6 Fig), using the same respiration and sensory input parameters as in Fig 7. ETCs are intrinsically entrained to periodic respiration-driven OSN inputs [36–38]. This rhythmic ETC system was previously thought to interpolate between sensory inputs and mitral cells, relaying input to the mitral cells [39]. Our addition of the cell to the network only included the excitatory synapses onto local and adjacent glomerulis’ PG cells (see Fig 7 in [36]) as the additional stimulation of MT cells is unlikely to have changed the network results. Although in our simulation the addition of ETC neurons did not strongly affect phase gating of sensory evoked excitation of the mitral cell, ETCs did slightly modulate mitral cell activity (compare S6 Fig to Fig 8). At lower stimulation levels (60–120 synaptic inputs), MTC activity was moderately increased in a phase-specific manner (10pi/6–0) by the addition of ETC (red line) to the network with PG inhibition (orange line in S6B Fig). At higher stimulation levels (S = 180–240), this increase was temporally less specific. The ETCs appeared to boost the MTC activity beyond the inhibition from respiration, either by relieving from or working against phase gating. Further investigation of these synapses *in vivo* will better reveal their roles in respiration mediated inhibition and gating of sensory input.

#### Respiration rate

**Fig 9:**
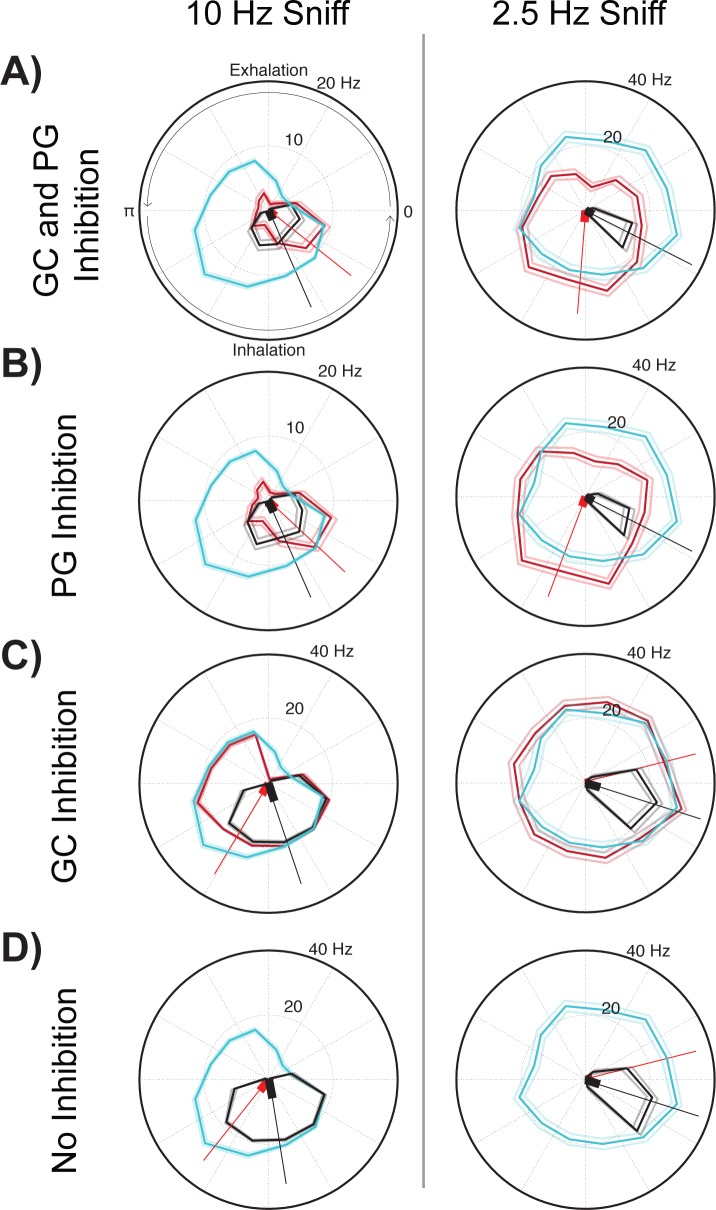
At increased sniff rates, granule and periglomerular inhibition temporally shift mitral cell activity across the respiratory cycle. A comparison of polar plots of mitral cell activity with sensory input (red, pink: ±SD), control without sensory input (black, grey: ±SD), and positive control with the same input in the absence of lateral inhibition (blue, light blue: ±SD). The circuit connectivity diagram used in this simulation is shown in Fig 7A. Both the respiratory and sensory input strengths were set to 300 excitatory synaptic events for all simulations in this figure. (A) Polar plots of data from the simulation with both periglomerular (PG) and granule cell (GC) inhibition present at a respiratory cycle rate of 10 Hz (left plot) and 2.5 Hz (right plot). Other figures are as (A), except in (B) only PG inhibition, in (C) only GC inhibition and in (D) no inhibition was present in the circuit model.

Next we used higher respiration rates to explore their influence on sensory evoked activity in mitral cells by periglomerular or granule cell inhibition. Using the same circuit (Fig 7), simulations were performed with a higher respiration rate to mimic typical active sniff rates (10 Hz, Fig 9). These were compared to the resting respiration rate that we previously examined (2.5 Hz, Fig 7, Fig 8 and S6 Fig). When respiratory and sensory evoked inputs were equal (300 excitatory sensory input events), higher respiration rates increased temporal coupling of sensory evoked activity with respiratory activity in the model with PG inhibition present (Fig 9A and 9B). With only GC inhibition present, centroid angles shifted to early phases of the respiratory cycle at higher respiration rates compared to lower respiration rates (Fig 9C). As this was also the case when no inhibition was present in the circuit model (Fig 9D), the refractory effect, not GC mediated inhibition, was responsible for this shift.

Simulations indicate that the respiration evoked and PG mediated inhibition play a prominent role in phase shifting mitral cell activity whereas respiration mediated GC inhibition is less pronounced. The variation in sniff frequencies can enhance and diversify the temporal gating of sensory evoked activity by PG and GC cells across a respiratory cycle. This ability of sniff frequency to modulate sensory input gating in mitral cells can occur with and without lateral inhibition present.

#### Lateral connectivity

The second variation of our simulation experiments used the circuit just described, except the number of connected glomerular columns (each containing a glomerular input, mitral, periglomerular and/or granule cell) was increased (see **glomeruli connectivity model** in Methods). Each connected glomerular column received respiratory inputs, but only the glomerulus synaptically connected to the recorded mitral cell received sensory input. All combinations of respiration and sensory input strengths were explored. This glomerular connectivity model was used to investigate how lateral inhibition stemming from multiple columns can influence respiration-mediated gating of mitral cell bulbar output [40].

**Fig 10:**
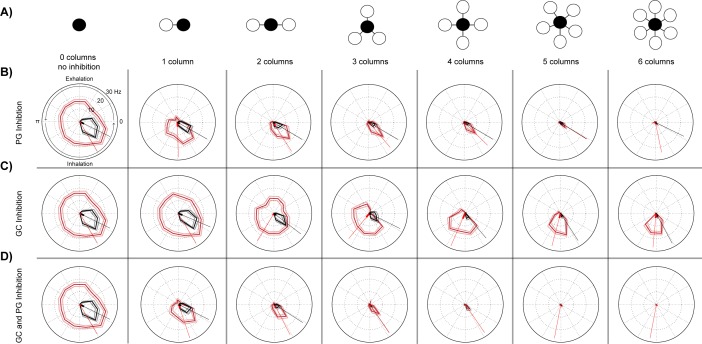
Increased glomerular column interconnectivity results in a temporal shift in evoked mitral cell activity across the respiratory cycle. (A) Simple column indicator icons describe the circuit model. Each black circle represents the column receiving respiratory and sensory evoked synaptic events, whereas all circles (white) receive only respiratory synaptic inputs. The 2 column model (second left diagram) contains the same circuitry as in (Fig 7A), whereas all of the other diagrams to the right have additional glomerular columns (white) connected with the recorded column (black). (B) Polar plots of mitral cell activity associated with each multiple column model with only periglomerular (PG) mediated inhibition (red, pink: ±SD) and control (black, grey: ±SD). (C) Same as (B) but with only granule cell (GC) inhibition present in the circuit model. (D) Similar to (B) but with both periglomerular and granule cell inhibition present. For all polar plots in the figure the respiratory inputs were comprised of 200 excitatory inputs and the additional sensory input was limited to only 100 synaptic events. Axis labels shown in the upper left polar plot are the same for all polar plots.

As the number of connected glomerular columns increased from 0 to 6 (Fig 10A), PGs and GCs (at a 30 times greater inhibitory input) inhibition shifted sensory evoked mitral cell activity. Shifts were seen with as few as one PG neuron connecting two glomeruli (Fig 10B), whereas with only GC inhibition gating required 2 or more connected glomerular columns (Fig 10C). With only GC inhibition, gating of sensory inputs occurred even after respiratory activity was nearly eliminated by lateral inhibition with 4 or more glomerular columns. This was not the case for the PG and the PG plus GC inhibition models (Fig 10D). With higher column connectivity, PG inhibition eliminated all mitral cell activity, both respiration and sensory driven. Increased glomerular column connectivity enhanced the role of GC inhibition in shaping mitral cell excitation across the respiratory cycle. Although GC inhibition did not play a prominent role in previous simulations with only two laterally connected glomerular columns, at higher levels of inter-glomerular connectivity, we began to see how GC cells, like PG cells, were also able to phase gate sensory evoked responses in mitral cells.

Under the PG-only inhibition condition, sensory and respiratory activity was nearly eliminated after 4 glomerular columns were connected. This was also true at lower respiratory (100 synaptic events) and sensory (150 synaptic events) inputs. PG neurons successfully gated activity to phases occurring before respiratory burst firing (S7 Fig, 1–4 columns), but in networks with higher glomerular connectivity all mitral cell activity was similarly eliminated. These data indicate that respiration mediated PG inhibition stemming from one or two glomerular columns strongly gate weaker excitatory inputs, whereas multiple glomerular columns connected by PG neurons can shunt both sensory evoked and respiration evoked phasic activity.

#### Secondary glomerular inputs

We also used the model to explore how secondary inputs to surrounding glomeruli alter the respiratory activity of the mitral cell. This model was used to explore inhibition by secondary glomerular inputs observed in our *in vivo* experiments (Figs 5 and 6). Here, a single glomerulus or multiple glomeruli were connected to the mitral cell by PGs and/or GCs (see methods, **glomeruli connectivity model)**. The connected glomeruli were activated by both respiratory and sensory inputs (grey glomerulus, see S8A Fig and **secondary glomerulus input model** in methods). The primary glomerulus synapsing onto the mitral cell recorded in the model only received respiratory inputs (white glomerulus, S8A Fig).

With only a single connected glomerular column, inhibition of respiratory activity in the recorded mitral cell by GC and/or PG neurons resulted in no significant shifts in centroid angle (a difference of < .25 radians, S8B Fig), centroid magnitude (a difference of < .4), or maximum firing rate (a difference of < 6 Hz). This was true across all stimulus and respiratory input strength combinations (20 to 720 synaptic events for both respiratory and sensory inputs). These simulation results indicate that the activation of a single glomerulus laterally connected to the recorded mitral cell does not influence the respiratory activity of the mitral cell.

However, when the number of glomerular columns connected to the recorded mitral cell increased, secondary inputs were able to reduce respiratory activity in the mitral cell, mimicking the inhibition of MTC activity observed *in vivo*. More than 3 interconnected glomerular columns were needed for PG neurons to abolish respiratory-evoked activity (S8B Fig, at an inhibitory input strength of 1 (black)), and more than 6 were needed for GCs to abolish respiration evoked activity. However, inhibition of respiratory activity was observed following a single glomerular column connection (at an inhibitory input strength of 4 (red dashed) and 20 (red) synapses S8B Fig). All respiration-evoked activity was inhibited when the mitral cell firing rates reached 2 Hz, which is the level of spontaneous activity set for the mitral cell in our simulations. The relative effectiveness of the different inhibitory networks (no inhibition, GC only, PG only, PG and GC) in enabling inhibition of respiratory activity by secondary sensory input mirrors the effectiveness of respiration mediated inhibition of primary glomerulus excitation (Fig 7B).

## Discussion

Our *in vivo* work demonstrates some of the first spatiotemporal maps of MTC receptive fields [18, 41–43] and importantly the corresponding temporal changes that occur across specific phases of respiration. We provide an effective method of subtracting respiration-coupled activity to isolate glomerular regions associated with evoking excitation and inhibition of MTCs. We focused on understanding how bursting MTCs are influenced by respiration during optical activation of sensory inputs.

Only 12% of mitral and 20% of tufted cells display burst firing that synchronizes with respiration [6]. In this study, respiratory activity in MTCs was found to be diverse in both its level and its degree of tuning to the transition from inhalation to exhalation (Fig 1 and [6, 17, 37, 42]). Respiration evokes neural rhythmic activity across species and has long been hypothesized to activate conserved mechanisms of odor processing [44, 45]. Even in the absence of respiration, oscillatory burst firing is observed in the antennal lobe circuit and is internally driven by inhibition [46–49]. Rhythmic firing in the bulb, either driven by respiration or spontaneously, strongly shapes sensory evoked circuit output responses. Sensory neuron evoked excitation of MTCs were influenced by the respiratory phase at which the stimulus was presented, which resulted in a net shift in their activity to earlier phases of the respiratory cycle (Fig 4C_4_). Optical stimulation of the same excitatory glomeruli never evoked MTC excitation during periods immediately following respiration mediated burst firing (Figs 2B, 2D, 3B, 3D, 4A_3_, 4B_3_ and 4C_1_). This refractory period may be used to temporally reset the olfactory network to receive the next series of odor inputs, analogous to discretization across breathing cycles. These findings suggest that respiration generates a temporal mechanism to gate sensory input excitation of MTCs. Entrainment of olfactory sensory neuron inputs with the respiratory cycle is not unique to rodents [50–52] and signifies a possible universal role of respiration in driving inhibition of sensory input during odor processing.

Using computational models we explored possible mechanisms to understand how respiration activates inhibition to gate sensory evoked excitation of MTCs. Respiration in our simulations activated inhibitory interneurons impinging on the MTCs. Respiration mediated phase-specific inhibition of sensory input was observed across physiological ranges of respiratory and sensory input strengths in the model (Fig 7B). In accord with our *in vivo* data, PG inhibition produced robust shifts in MTC activity following excitation by a wide range of sensory and respiration input strengths.

It is important to note that our PG cell model was simplified. It contained both inter- and intra-glomerular inhibitory synapses, to allow our findings to be generalized to several subtypes of periglomerular cells and short axon cells [31]. There is much diversity in the different juxtaglomerular interneurons in the glomerular layer circuit [31, 35, 53, 54] and inter- and intra-glomerular inhibition likely play distinct roles in sensory processing. With our simulation, we find that sensory evoked phase shifts in MTC activity are predominantly mediated by intraglomerular inhibition (S5 Fig), whereas the addition of ETCs appear to only moderately modulate the strength of tuning (S6 Fig). However, interglomerular inhibition and/or ETC input may be important for processing other odor information, such as intensity [43].

Excitation by respiration alone, in the absence of any lateral inhibition, was also able to weakly gate mitral cell activity, i.e. show a refractory period (Fig 7B yellow/black lines, no inhibition present condition and S3E Fig). This weak shift in mitral cell activity across the respiratory cycle was similar to that seen with physiologically relevant levels of granule cell inhibition (4–30 synaptic contacts per lateral dendrite) at low respiration frequencies. This indicated that GC inhibition may not be a strong mediator of phasic MTC activity at low respiration rates.

As respiration rates increase during periods of investigation, mitral cell and interneuron activity has been shown to decouple with each respiratory cycle and filter sensory inputs [7, 55–58]. In our simulations, we found that *increased coupling to respiration* could also occur, but only when sensory inputs were very strong and overrode all phasic respiratory activity in MTCs. Indeed, when strong sensory inputs evoked responses during all phases of the respiratory cycle, we found that at higher sniff rates the coupling of MTC activity to respiration increased, especially so when periglomerular inhibition was present (Fig 9A and 9B). This has interesting implications when considering how the olfactory bulb system might override strong odor inputs to focus on weaker stimuli of interests.

Phase gating only slightly increased with GC inhibition at high respiration rates (Fig 9C). However, when GC inhibition was driven by a lower rate of respiration, and three or more glomerular columns entrained to the respiratory cycle were laterally connected 1) sensory input evoked excitatory responses were gated to earlier phases of the respiratory cycle and 2) all respiratory burst firing was nearly or entirely abolished (Fig 9C). High intensity odor stimuli activate larger populations of inhibitory GC *in vivo* [59] and can theoretically impinge onto the lateral dendrites of a single MTC [40]. GCs also displayed patterns of activity associated with respiration [8, 60]. According to our simulations, if many GCs could be recruited by respiration then GC inhibition may be able to modulate respiratory evoked activity itself. The ability for GC inhibition to selectively modulate respiratory activity without eliminating the gating of sensory evoked responses could increase the odor dependent signal to noise ratio by separating sensory and spontaneous respiratory inputs, which may have interesting implications for GC inhibition during odor discrimination [61].

In contrast, this ability to modulate respiratory evoked responses in isolation was not evident for the multicolumn periglomerular inhibition models. Increasing the PG lateral inhibitory connectivity resulted in the inhibition of all sensory and respiration evoked firing by the mitral cell (Fig 10B, last two columns, S7 Fig). These findings may imply the existence of sparse and synchronized patterns of MTC activity in the bulb [62, 63], since the phase gating of sensory input responses that we found *in vivo* was mainly observed *in silico* when only few interconnected glomerular columns were activated. Although increasing respiration rates had a much stronger effect on PG phase gating of mitral cells (Fig 9A) compared to equivalent inputs observed in the GC inhibition model (Fig 9C), increasing connectivity heightened the role of GC inhibition and abolished the role of PG inhibition.

High respiratory rates, at levels seen in actively sniffing mice, enhanced the temporal gating of mitral cell activity across the respiratory cycle. In the case of periglomerular inhibition, the higher respiratory rate increased the coupling of sensory evoked activity to respiration-mediated burst firing. In contrast, when examining granule cell mediated inhibition or no lateral inhibition, mitral cell activity shifted to earlier phases of the respiratory cycle (Fig 9) at higher sniff rates, but there was no increased coupling between sensory and respiratory evoked responses compared to low breathing rates. These mechanisms of sensory input gating are likely functionally relevant as awake mice detect differences in the temporal dynamics of mitral and tufted cell activity at a fast 13 ms resolution [64]. The ability of respiration to affect lateral inhibition may therefore play a significant role in odor detection and recognition. It will be of interest to explore how sensory input may interact with the phasic preferences of mitral and tufted cells, as well as interneuron activity across varying respiratory frequencies in awake mice.

GC mediated phase gating was more subtle than and overridden by PG inhibition. The periglomerular synapses reside immediately below the initial bifurcation of the apical tuft dendrites of the mitral cell [65]. This anatomical feature enables the periglomerular inhibitory synapse to interact with the sensory inputs in the apical tuft (Fig 7A) of the mitral cell *before* the excitatory event propagates to the soma and lateral dendrites where it can interact with granule cells. The simulated network conditions that examined gating of MTC activity suggest a primary role for PG cells and a secondary role for granule cells in phase gating MTC output. These conclusions corroborate previous work suggesting that non-specific gain control of sensory input is mediated by PG inhibition and sparse yet specific sensory input processing can be controlled by GC inhibition [66, 67]. All these three mechanisms—PG inhibition, GC inhibition, and the respiration evoked refractory period—can contribute to temporal gating of sensory input in the olfactory bulb.

Simulations of secondary glomerular mediated inhibition (S8B Fig), with both periglomerular and granule cell lateral inhibition, produced mitral cell responses similar to those observed *in vivo* (Figs 5 & 6), but only when multiple glomerular columns were connected via granule or periglomerular interneuron synapses. Although our model provides a possible mechanism, recent work has found that direct optical stimulation of mitral cells can reduce the membrane potential of neighboring mitral cells. This reduction in the excitable properties of MTCs is likely mediated by GC inhibition and not a result of recurrent or delayed self-inhibition [41].

The work presented here has revealed the temporal interactions between respiration- and sensory-evoked lateral inhibition, which can form the basis to further explore how spatio-temporal inhibition patterns in the bulbar circuit shapes MTCs output. The activity of glomeruli and MTCs across the respiratory cycle is believed to shape odor perception [14, 61, 68, 69]. This work has interesting implications for how the sniff rate may gate MTC output to sensory inputs, specifically as higher sniff rates increase inhibition [70, 71] and tighten mitral and tufted cell activity across the respiratory cycle [72]. Respiration not only distributes odors across the nasal epithelium, but also temporally shapes lateral inhibition and mitral cell burst firing in the bulb.

## Supporting Information

S1 Fig Diagram of the optical stimulus paradigm. **(A)** A schematic of a series of 5 images that were similar to the ~3000–5000 images that were projected onto the dorsal surface of that olfactory bulb during each MTC extracellular recording. Single blocks of light were individually projected onto unique regions of the dorsal OB to avoid repeatedly stimulating the same site. **(B)** For each light block stimulation a corresponding block was added to a heatmap and was colored to match the number of spikes that were recorded during that stimulation. Heatmap blocks that overlapped were averaged in the areas in which they overlapped (see fifth heatmap from top and corresponding inset that zooms into an area where stimulation blocks previously overlapped).
(TIF)

S2 Fig Computational model input strengths. Inputs to the models for both the respiration and light stimulus were modeled as synaptic events in the olfactory sensory neurons generated by Gaussians of Poisson distributed processes. The Gaussian peaks were varied while their half widths were always 30 milliseconds. **(A)** The table gives the average number of spikes these Gaussians generate per respiration and their overall firing rate when the respiration cycle frequency is 2.5 Hz (2.5 respirations per second). For alignment, the simulated respiration's Gaussian peaks were positioned at polar angle 0 and for the light stimulus the rising (left) half width from the peak was assigned to the onset time of the modeled light stimulus. In the results, synaptic input values are stated as the average number of excitatory inputs, which is the peak value of a Gaussian input. **(B)** The Gaussians for the values provided in the table are shown in the graph.
(TIF)

S3 Fig Inhibition of sensory evoked excitation following burst respiratory firing. **(A)** Example polar plot data of stimulated (red, pink: ±SE) and control (black, grey: ±SE) activity from a simulation with periglomerular and granule cell inhibition. **(B)** Diagram of circuit with neurons colored to match corresponding traces below. Color-coded voltage traces of activity recorded from the soma of each neuron in the model from cycle angles 1.5/π (left column of traces) and 7.5/π (right column of traces) indicated with black arrows in **(C)** are examined with only periglomerular inhibition, **(D)** only granule cell inhibition (30 synaptic contacts), and **(E)** with no inhibition present.
(TIF)

S4 Fig Polar plots comparing periglomerular and mitral cell activity with and without stimulation. Three polar plots of neuronal activity across the respiratory cycle corresponding to the circuit diagram in Fig 7A. Respiration was set to produce 200 excitatory inputs and the sensory input was varied to produce 60, 120, and 180 excitatory inputs. Black line (grey = ±SE): MTC activity without sensory input stimulation. MTC (red, pink: ±SE) and PG (orange, light orange: ±SE) activity during sensory and respiration input stimulation. Blue line (light blue = ±SE): MTC activity with both sensory and respiratory input in the absence of lateral inhibition.
(TIF)

S5 Fig Comparison of intra- and inter-periglomerular inhibition. Three simulations were performed where **(A)** both lateral and reciprocal PG synapses were intact (same as in Fig 7), **(B)** the interglomerular lateral PG synapse was removed, or **(C)** the reciprocal intraglomerular PG synapse was removed. Below these models are their corresponding polar plots in **(D, E, F).** Respiration was set to produce 200 excitatory inputs and the sensory input was varied to produce 120, 180, and 240 excitatory inputs. Red lines (pink lines = ±SD) are of mitral cell activity in the simulation where PG inhibition is present. Blue lines (light blue lines = ±SD) are of mitral cell activity in the simulation without network inhibition. Black lines (grey lines = ±SD) are responses of the mitral cell when only respiration is present in the absence of sensory input.
(TIF)

S6 Fig Addition of external tufted cells did not affect phase gating of sensory evoked responses. **(A)** A simple circuit diagram of the neural model with external tufted cells (ETC). **(B)** Polar plots of stimulated (red, pink: ±SE) and control non-simulated (black, grey: ±SE) conditions with all synaptic connections as shown in (A) but without GC inhibition. Orange polar plots are taken from Fig 8A to allow for a comparison of stimulated MTC responses with ETCs (red, pink ±SE) and without ETCs (orange, light orange ±SE). Blue lines (light blue lines = ±SD) are of mitral cell activity in the simulation without network inhibition. Respiratory inputs for each plot are set to 200 and stimulation inputs are varied from 60 to 240, as shown in the ratio above each plot (respiratory input: stimulus input). Radii (y-axis) scale and respiratory cycle angles (radians) shown in the upper left polar plot is the same for all polar plots. **(C)** Same as panel (B), but with the addition of GC inhibition, exactly as seen in the circuit diagram in panel (A). Notice there is no change in MTC responses (red, pink ±SE) to sensory input with (C) or without GC inhibition (B).
(TIF)

S7 Fig Increased glomerular column interconnectivity allows periglomerular inhibition to effectively shift mitral cell activity in response to small sensory inputs. (A) Diagrams of circuits corresponding to their polar plots in B. As in Fig 9, the black circle represents the column receiving the additional sensory synaptic events, whereas the white circles represent the connected columns that are receiving only synchronous respiratory inputs. (B) Polar plots of mitral cell activity associated with each multiple column model with only periglomerular cell (PG) mediated inhibition (red, pink: ±SD) and control (black, grey: ±SD). For all polar plots in the figure the respiratory inputs were comprised of 100 excitatory inputs and the additional sensory input was limited to only 150 synaptic events. Axis labels shown in the upper left polar plot are the same for all polar plots.
(TIF)

S8 Fig Lateral inhibition decreases respiratory activity when multiple secondary glomeruli are activated. (A) Schematic of the circuit model. One recording is obtained from one mitral cell (black, left). This neuron received only respiratory inputs at its glomerulus. A second mitral cell (grey) receives both respiratory and additional sensory inputs (mimicking excitation by a secondary glomerulus). The number of connected secondary glomeruli is increased (as in Fig 9). (B) As the number of connected glomeruli was increased, inhibition of respiratory activity also increased, either by granule cells (synaptic weights 4 (dotted red) and 20 (solid red)) or by periglomerular neurons (black).
(TIF)
