# Supplementary figures and images for: Respiration Gates Sensory Input Responses in the Mitral Cell Layer of the Olfactory Bulb
Source: PLoS One. 2016 Dec 22;11(12):e0168356. doi: 10.1371/journal.pone.0168356 (PMC5179112; doi:10.1371/journal.pone.0168356)

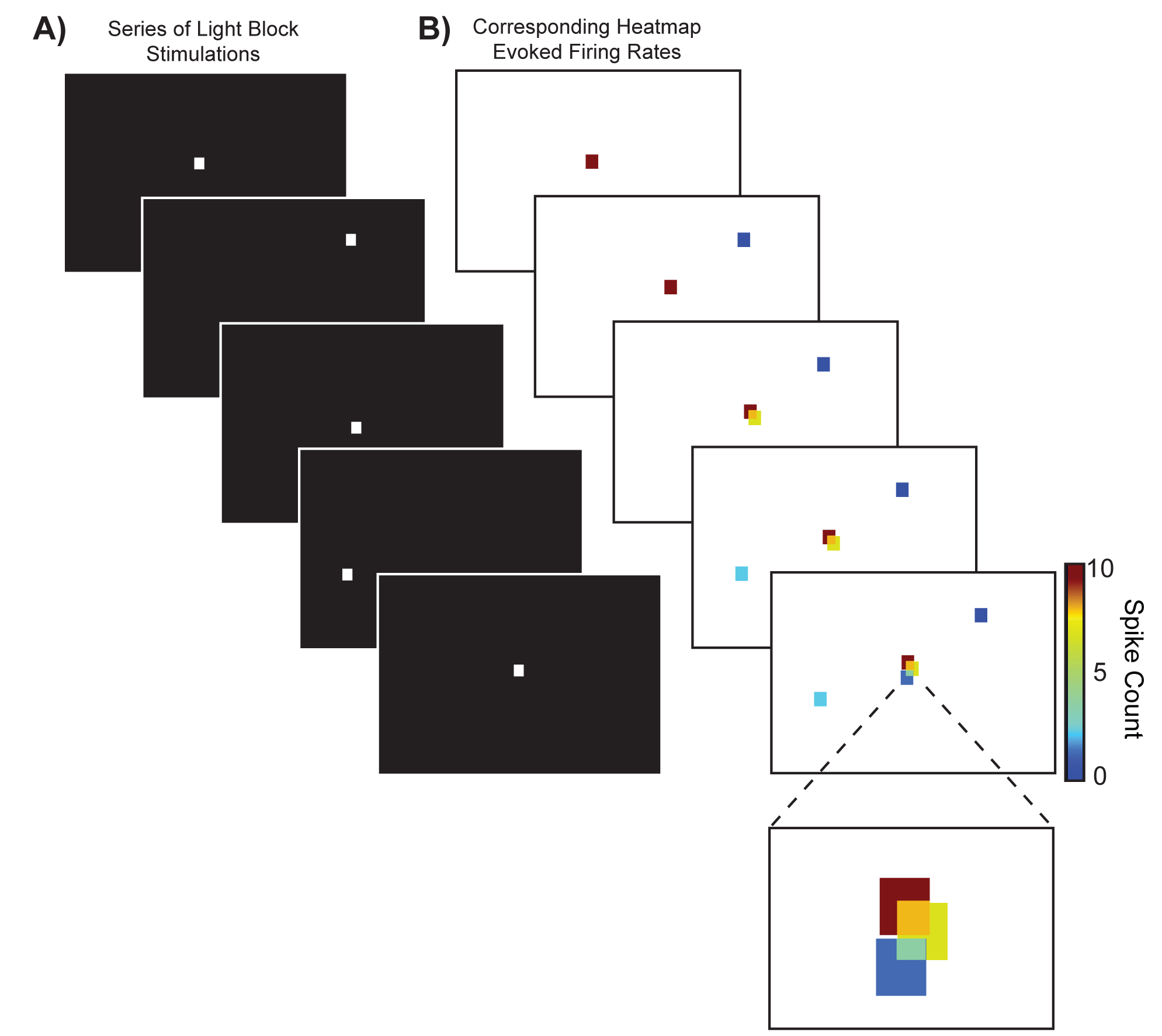

Supplement: S1 Fig — (A) A schematic of a series of 5 images that were similar to the ~3000–5000 images that were projected onto the dorsal surface of that olfactory bulb during each MTC extracellular recording. Single blocks of light were individually projected onto unique regions of the dorsal OB to avoid repeatedly stimulating the same site. (B) For each light block stimulation a corresponding block was added to a heatmap and was colored to match the number of spikes that were recorded during that stimulation. Heatmap blocks that overlapped were averaged in the areas in which they overlapped (see fifth heatmap from top and corresponding inset that zooms into an area where stimulation blocks previously overlapped). (TIF) [file pone.0168356.s001.tif]

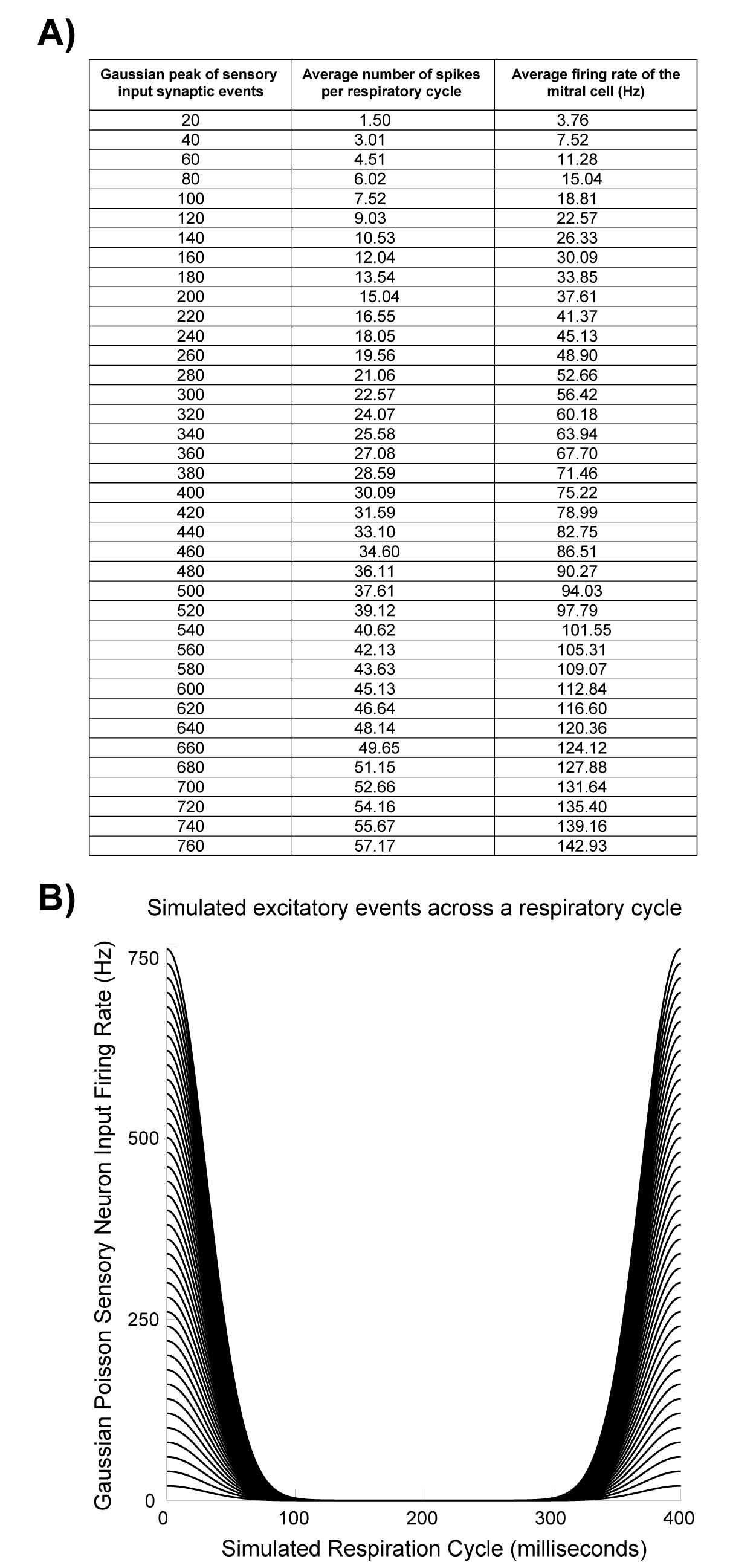

Supplement: S2 Fig — Inputs to the models for both the respiration and light stimulus were modeled as synaptic events in the olfactory sensory neurons generated by Gaussians of Poisson distributed processes. The Gaussian peaks were varied while their half widths were always 30 milliseconds. (A) The table gives the average number of spikes these Gaussians generate per respiration and their overall firing rate when the respiration cycle frequency is 2.5 Hz (2.5 respirations per second). For alignment, the simulated respiration's Gaussian peaks were positioned at polar angle 0 and for the light stimulus the rising (left) half width from the peak was assigned to the onset time of the modeled light stimulus. In the results, synaptic input values are stated as the average number of excitatory inputs, which is the peak value of a Gaussian input. (B) The Gaussians for the values provided in the table are shown in the graph. (TIF) [file pone.0168356.s002.tif]

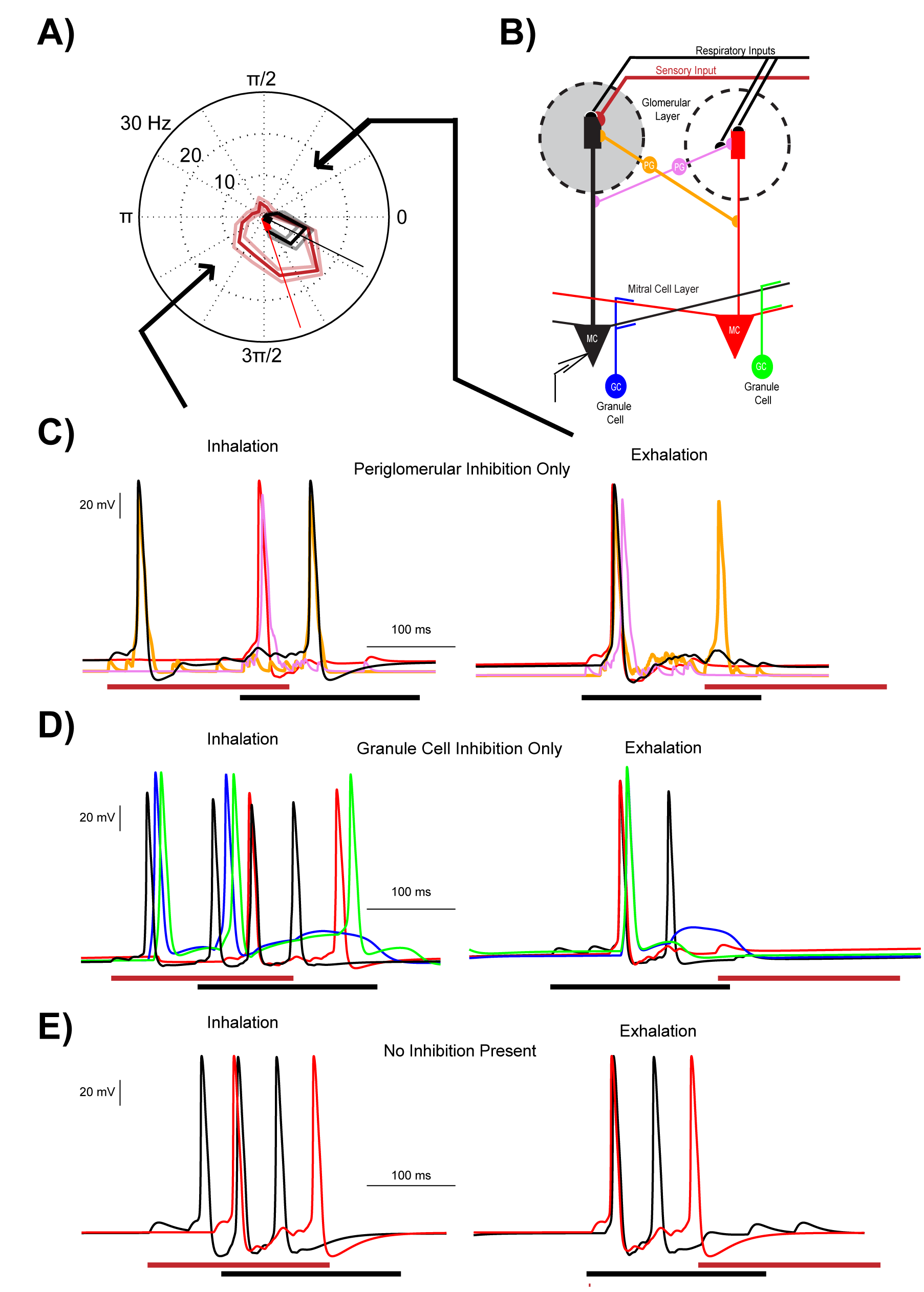

Supplement: S3 Fig — (A) Example polar plot data of stimulated (red, pink: ±SE) and control (black, grey: ±SE) activity from a simulation with periglomerular and granule cell inhibition. (B) Diagram of circuit with neurons colored to match corresponding traces below. Color-coded voltage traces of activity recorded from the soma of each neuron in the model from cycle angles 1.5/π (left column of traces) and 7.5/π (right column of traces) indicated with black arrows in (C) are examined with only periglomerular inhibition, (D) only granule cell inhibition (30 synaptic contacts), and (E) with no inhibition present. (TIF) [file pone.0168356.s003.tif]

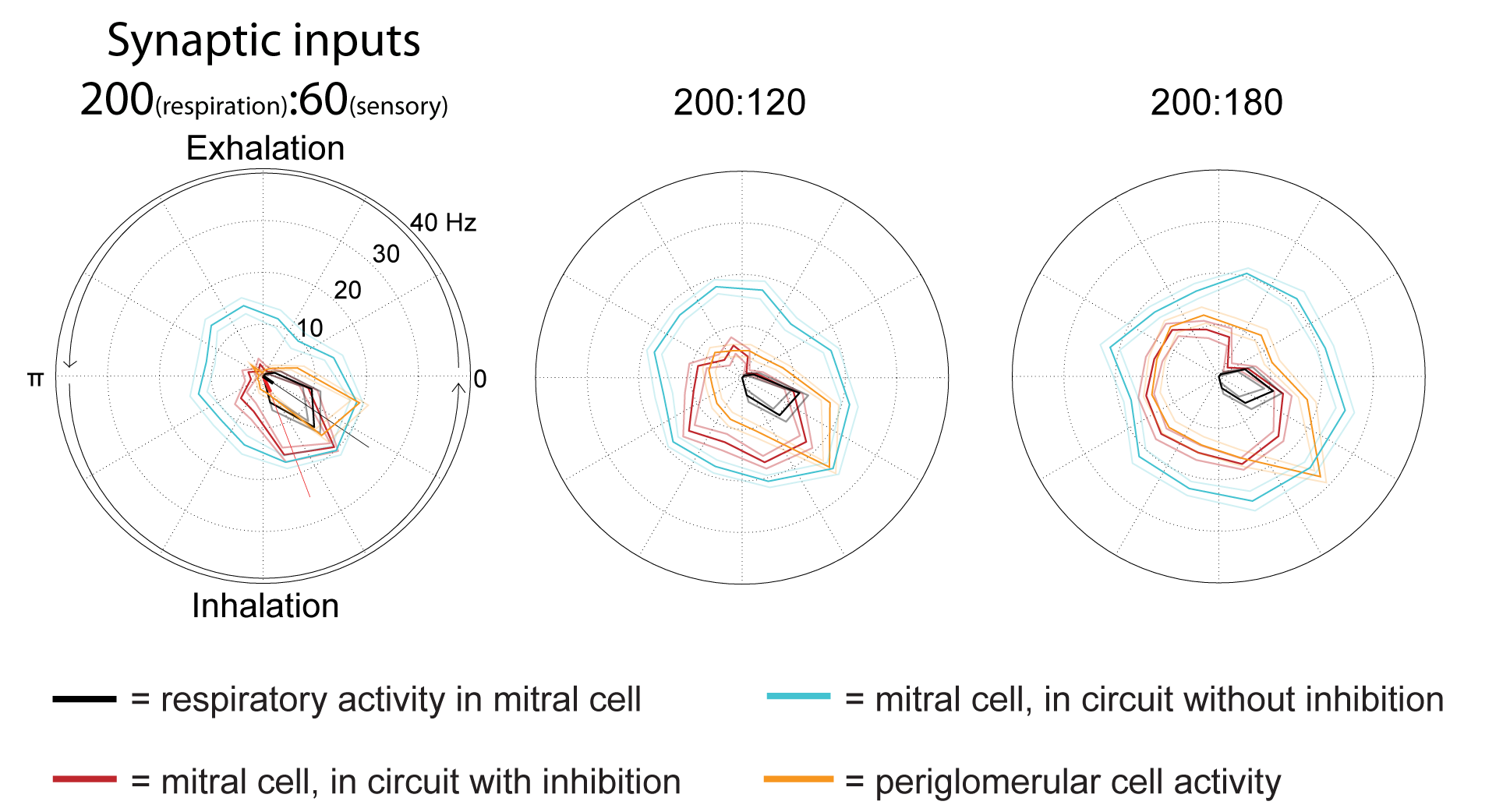

Supplement: S4 Fig — Three polar plots of neuronal activity across the respiratory cycle corresponding to the circuit diagram in Fig 7A. Respiration was set to produce 200 excitatory inputs and the sensory input was varied to produce 60, 120, and 180 excitatory inputs. Black line (grey = ±SE): MTC activity without sensory input stimulation. MTC (red, pink: ±SE) and PG (orange, light orange: ±SE) activity during sensory and respiration input stimulation. Blue line (light blue = ±SE): MTC activity with both sensory and respiratory input in the absence of lateral inhibition. (TIF) [file pone.0168356.s004.tif]

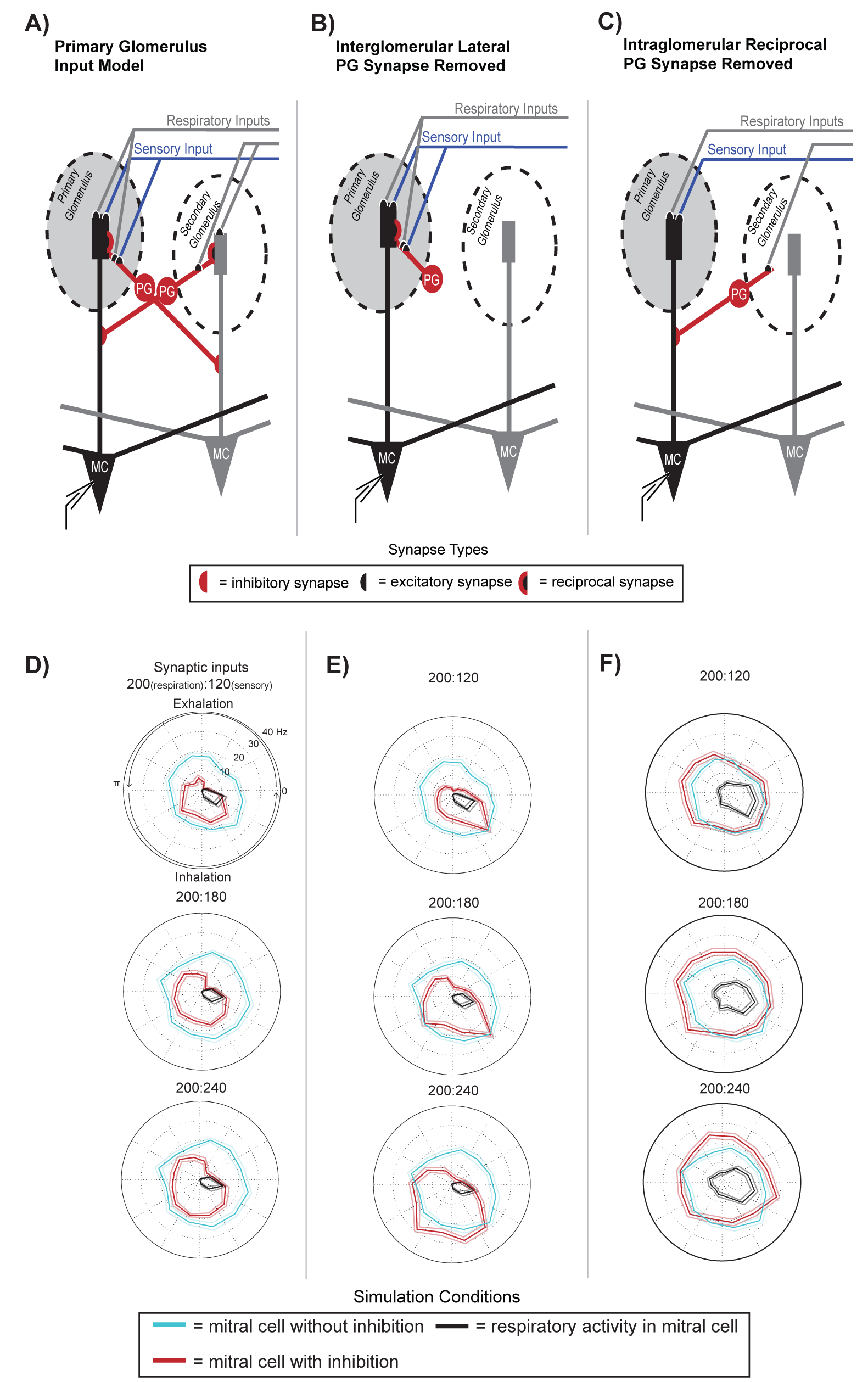

Supplement: S5 Fig — Three simulations were performed where (A) both lateral and reciprocal PG synapses were intact (same as in Fig 7), (B) the interglomerular lateral PG synapse was removed, or (C) the reciprocal intraglomerular PG synapse was removed. Below these models are their corresponding polar plots in (D, E, F). Respiration was set to produce 200 excitatory inputs and the sensory input was varied to produce 120, 180, and 240 excitatory inputs. Red lines (pink lines = ±SD) are of mitral cell activity in the simulation where PG inhibition is present. Blue lines (light blue lines = ±SD) are of mitral cell activity in the simulation without network inhibition. Black lines (grey lines = ±SD) are responses of the mitral cell when only respiration is present in the absence of sensory input. (TIF) [file pone.0168356.s005.tif]

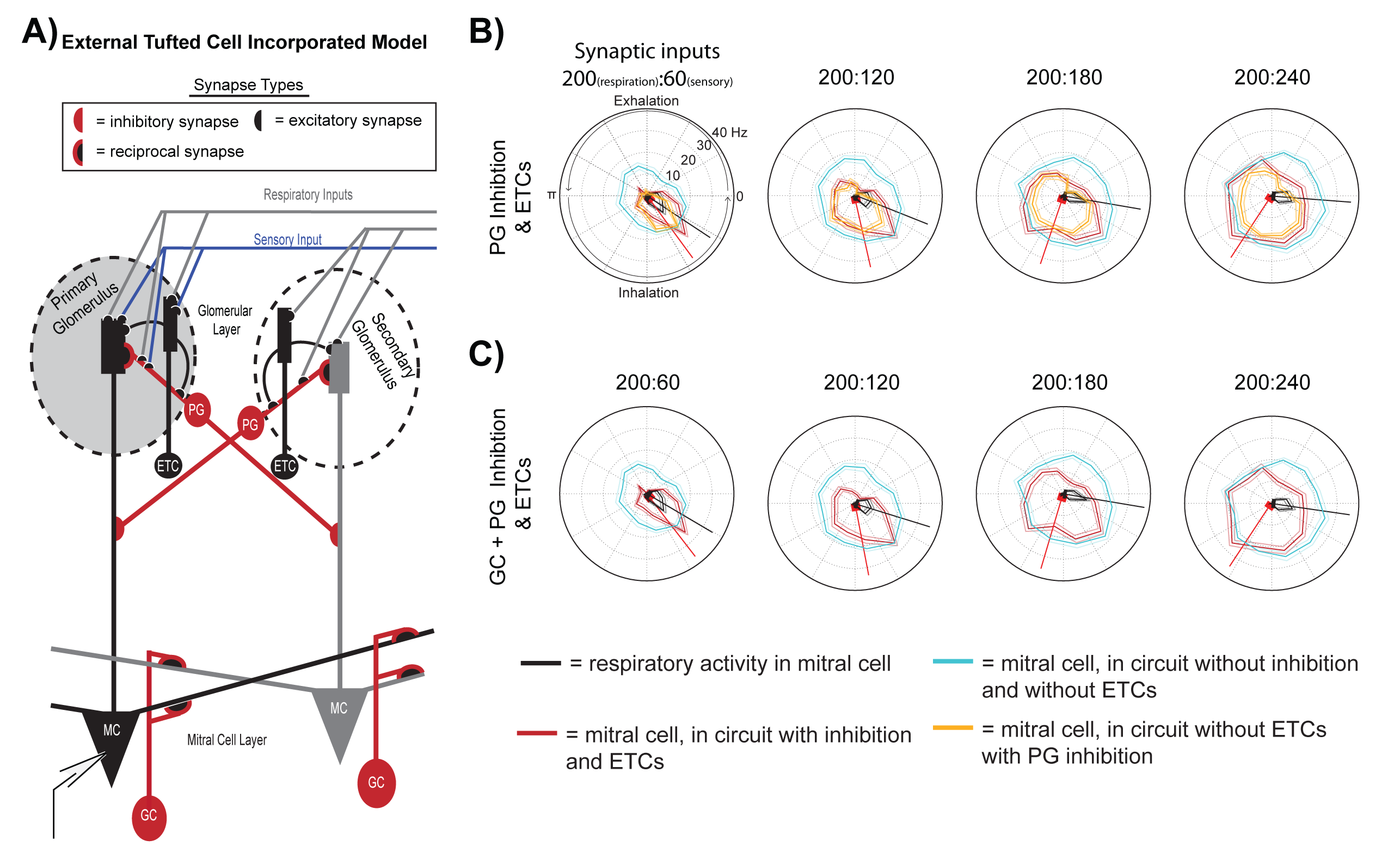

Supplement: S6 Fig — (A) A simple circuit diagram of the neural model with external tufted cells (ETC). (B) Polar plots of stimulated (red, pink: ±SE) and control non-simulated (black, grey: ±SE) conditions with all synaptic connections as shown in (A) but without GC inhibition. Orange polar plots are taken from Fig 8A to allow for a comparison of stimulated MTC responses with ETCs (red, pink ±SE) and without ETCs (orange, light orange ±SE). Blue lines (light blue lines = ±SD) are of mitral cell activity in the simulation without network inhibition. Respiratory inputs for each plot are set to 200 and stimulation inputs are varied from 60 to 240, as shown in the ratio above each plot (respiratory input: stimulus input). Radii (y-axis) scale and respiratory cycle angles (radians) shown in the upper left polar plot is the same for all polar plots. (C) Same as panel (B), but with the addition of GC inhibition, exactly as seen in the circuit diagram in panel (A). Notice there is no change in MTC responses (red, pink ±SE) to sensory input with (C) or without GC inhibition (B). (TIF) [file pone.0168356.s006.tif]

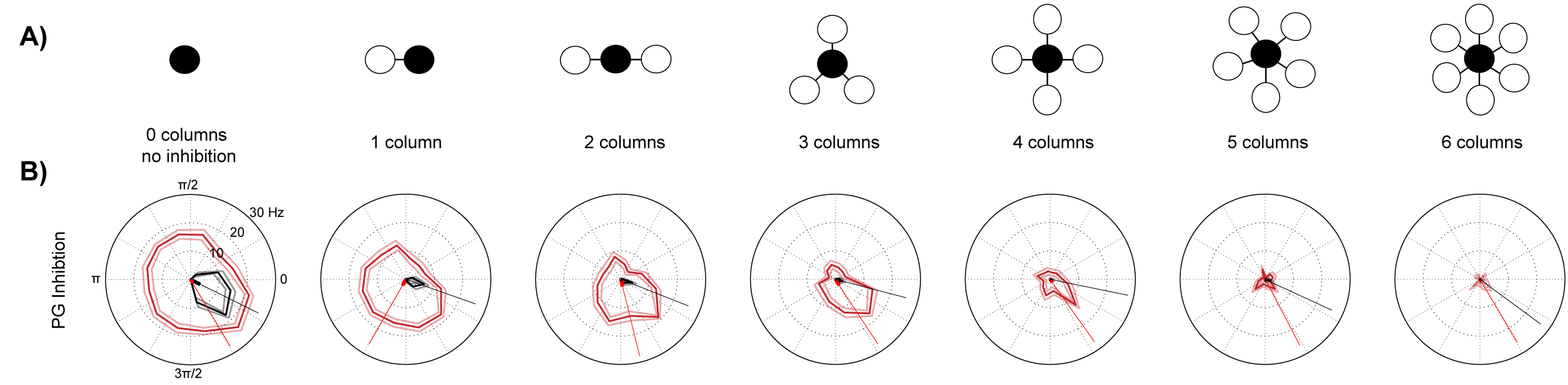

Supplement: S7 Fig — (A) Diagrams of circuits corresponding to their polar plots in B. As in Fig 9, the black circle represents the column receiving the additional sensory synaptic events, whereas the white circles represent the connected columns that are receiving only synchronous respiratory inputs. (B) Polar plots of mitral cell activity associated with each multiple column model with only periglomerular cell (PG) mediated inhibition (red, pink: ±SD) and control (black, grey: ±SD). For all polar plots in the figure the respiratory inputs were comprised of 100 excitatory inputs and the additional sensory input was limited to only 150 synaptic events. Axis labels shown in the upper left polar plot are the same for all polar plots. (TIF) [file pone.0168356.s007.tif]

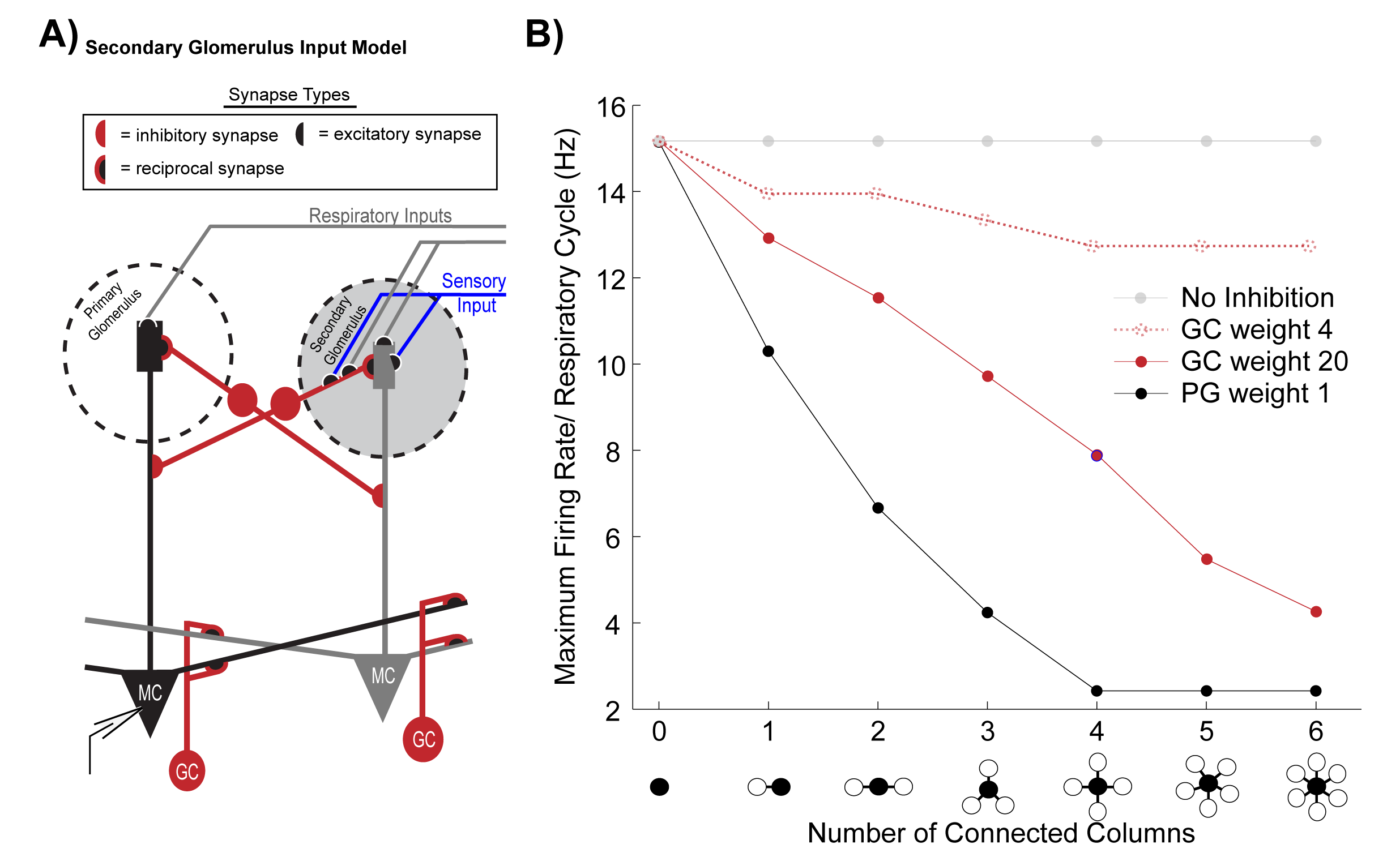

Supplement: S8 Fig — (A) Schematic of the circuit model. One recording is obtained from one mitral cell (black, left). This neuron received only respiratory inputs at its glomerulus. A second mitral cell (grey) receives both respiratory and additional sensory inputs (mimicking excitation by a secondary glomerulus). The number of connected secondary glomeruli is increased (as in Fig 9). (B) As the number of connected glomeruli was increased, inhibition of respiratory activity also increased, either by granule cells (synaptic weights 4 (dotted red) and 20 (solid red)) or by periglomerular neurons (black). (TIF) [file pone.0168356.s008.tif]
